# Polymeric Electrospun Nanofiber Composites as Fast Equilibrium Passive Samplers: Integration of Surface Functionalities and Porosity to Improve Organic Chemical Uptake

**DOI:** 10.1021/acsenvironau.5c00183

**Published:** 2025-12-11

**Authors:** Matthew R. Nagorzanski, Jiajie Qian, Sarah A. Crane, David M. Cwiertny, Andres Martinez

**Affiliations:** †Department of Civil & Environmental Engineering, IIHR—Hydroscience & Engineering, ‡Department of Chemical & Biochemical Engineering, §Department of Chemistry, The University of Iowa, Iowa City, Iowa 52246, United States

**Keywords:** passive sampling, electrospinning, carbon nanotubes, surfactant, pesticides, fast uptake, reversibility

## Abstract

Despite advances in passive sampling technologies, challenges persist in improving the selectivity, sensitivity, and response time. This study presents the fabrication and evaluation of electrospun nanofiber mats (ENMs) embedded with carbon nanotubes (CNTs), with and without surfactant modifications, as fast equilibrium passive sampling materials. We investigated the sorption and desorption behaviors of four common surface water contaminants: atrazine, metolachlor, diuron, and 2,4-dichlorophenoxyacetic acid (2,4-D). We demonstrated that ENMs modified with the cationic surfactant tetrabutyl ammonium bromide (TBAB) exhibited higher sorption than their unmodified PAN/CNT counterparts for all species, including anionic 2,4-D, for which uptake increased by up to 45-fold. ENMs modified with sodium dodecyl sulfate (SDS), a leachable porogen, exhibited greater surface area and improved sorption of atrazine, metolachlor, and diuron, resulting in an 8- to 40-fold increase in uptake. Across formulations, sorption to CNT-containing ENMs was largely reversible, with stronger, more irreversible binding at higher CNT wt %. The optimal formulation of PAN/10 wt % COOH-CNT/20 wt % SDS exhibited rapid, reversible sorption of atrazine, with 96% desorption after 48 h and fast equilibrium in response to changing solution concentrations. Field deployment in an agriculturally impacted creek showed good agreement with grab samples for atrazine (with ENM-derived concentrations within 5–40% of grab sample-derived concentrations) but overestimated metolachlor concentrations (with ENM-derived concentrations up to 500% greater than grab sample-derived concentrations), which we attribute to metolachlor's greater hydrophobicity, resulting in more irreversible binding to CNTs. Although further refinement is needed, these findings highlight the potential of ENM-CNT composites as novel materials for use as fast equilibrium passive samplers, especially for atrazine, and underscore the importance of tailoring the ENM composition to target specific micropollutants.

## Graphical abstract

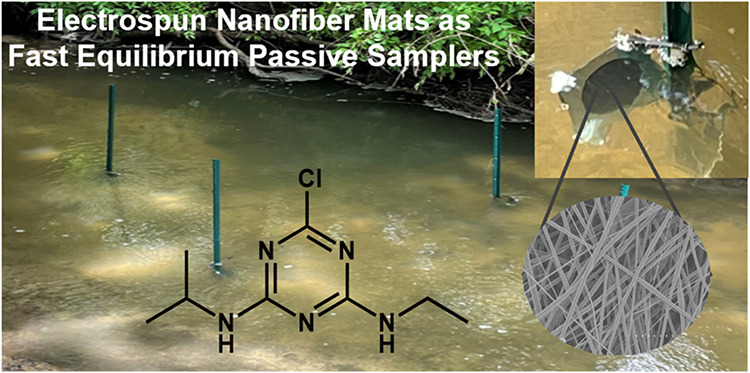

## Introduction

Recent advances in passive sampling devices have focused on new materials with physical and chemical properties that enable application to more diverse chemical suites.^1,2^ For example, the Polar Organic Chemical Integrative Sampler (POCIS) uses materials that target more polar, higher water solubility chemicals (log *K*_ow_ < 3) such as pharmaceuticals and pesticides. POCIS relies on commercially available but proprietary functionalized resins (e.g., Isolute ENV+ and Ambersorb 1500, or Oasis HLB) commonly used for solid-phase extraction to sorb these more polar compounds.^3^ Similarly, diffusive gradients in thin films (DGT) samplers can be adapted with specific binding layers to capture certain polar organic compounds, providing an alternative approach for hydrophilic chemicals in aquatic environments.^4^

Despite these advances, passive sampling materials with greater capacities and faster uptake rates remain desirable because they would allow for more sensitive quantification over shorter deployment time scales for certain high-priority pollutant classes. Low detection levels are particularly important for polar pharmaceutical and pesticide chemical classes typically targeted by POCIS whose surface water concentrations often range from tens to thousands of ng/L.^5–9^ Although low (at parts per trillion levels), these exposures are still a concern for the ecosystem, and in some cases, human health.^7,10,11^

Another challenge lies in the effective sampling of charged analytes.^12–14^ Many high-priority pollutants are either permanently charged or possess ionizable groups with p*K*_a_ values relevant to surface waters (∼6–9), making them partially or fully ionized in environmental systems. Examples include 2,4-dichlorophenoxyacetic acid (2,4-D) and glyphosate, pesticides regulated under the U.S. Safe Drinking Water Act.^15–17^ Due to their charge and resulting high water solubility, it is unclear whether traditional passive samplers (e.g., POCIS) designed for neutral compounds are effective for these analytes.^14^

Our previous work showed that electrospun nanofiber mats (ENMs) and nanocomposites offer key advantages for passive sampling with tunable material properties critical to performance. Their high surface area-to-volume ratio enables rapid micropollutant uptake.^18^ Incorporating carbon nanotubes (CNTs) produces polymer-CNT composites with enhanced uptake rates and capacities, likely from sorption on CNTs near the fiber surface.^19–21^ Although reversibility was not fully examined, these composites behaved as fast equilibrium samplers and showed good agreement with grab samples when deployed as a proof-of-concept for atrazine monitoring in an agriculturally impacted creek.^20^

Surfactants are commonly used in electrospinning to improve fiber formation by increasing conductivity, resulting in more uniform nanofiber morphology and tighter diameter distributions.^22–27^ Some surfactants, including quaternary ammonium salts (QAS), can also migrate to the polymer–air interface during synthesis, enriching the fiber surface with charged groups that provide useful chemical functionality, such as ion exchange capacity.^19,28^ Surfactants can also act as porogens: their intentional removal after fabrication creates cavities in the polymer matrix, increasing pore volume and specific surface area of the fibers.^22,29–31^ These properties enhance the versatility of electrospun nanofibers for passive sampling applications.

Here, we expand on our work with ENMs for passive sampling^18,20^ by developing surfactant-functionalized, polymer-CNT composites with enhanced uptake of organic micropollutants, including charged species. QAS (e.g., tetrabutyl ammonium bromide or TBAB) were incorporated during synthesis to increase surface positive charge and improve affinity for electron-rich and anionic pollutants. Sodium dodecyl sulfate (SDS) was used as a porogen to increase composite surface area and pore volume, whereas any residual SDS may also enhance the uptake of positively charged species via electrostatic interactions.

To advance their use in passive sampling applications, we then examined the sorption–desorption behavior of atrazine and metolachlor on the most promising surfactant-functionalized polymer-CNT composites and evaluated the material response to changing solution concentrations during performance trials. These studies assessed the thermodynamic reversibility of chemical uptake, a key requirement for equilibrium passive sampling, and informed models for estimating surface water micropollutant concentrations from equilibrium binding data. Finally, we conducted another proof-of-concept field deployment targeting atrazine and metolachlor in an agriculturally impacted stream, Muddy Creek in North Liberty, IA, to evaluate the material performance as surface water passive sampling devices.

## Material and Methods

### Reagents

A complete reagent list is in the Supporting Information (SI). ENMs were fabricated from polyacrylonitrile (PAN; MW ∼150,000; Sigma-Aldrich). Nonfunctionalized carbon nanotubes (NF-CNTs) and carboxylic acid functionalized carbon nanotubes (COOH-CNTs) are described in SI (Table S1). Surfactants used in ENM composites were TBAB and SDS (Table S2). Target analytes were atrazine, 2,4-D, metolachlor, and diuron (Table S3).

### Electrospinning

ENMs were synthesized using established methods^19,20,28,29^ typically incorporating surfactants and CNTs (NF-CNT or COOH-CNT) each at loadings of 20 wt % relative to PAN (some formulations were prepared at 10 wt %). For SDS-treated ENMs, a postsynthesis leaching step was applied to enable SDS’s role as a porogen.^29^ Hereafter, we use a nomenclature for different formulations in which the wt % (relative to PAN) of various ingredients in the electrospinning sol gel are noted as subscripts (e.g., PAN/TBAB_20_/COOH-CNT_20_ contains TBAB and COOH-CNTs each at 20 wt %). Detailed synthesis conditions are provided in Table S4.

### Material Characterization

Nanofiber morphology and diameter distributions were determined using a Hitachi S-4800 scanning electron microscope (SEM) with image processing using ImageJ (freely available through the NIH). The specific surface area and total pore volume of ENMs were measured by using N_2_-BET adsorption isotherms. Additional chemical characterization of nanofibers was conducted using Attenuated Total Reflectance Fourier Transform Infrared Spectroscopy (ATR-FTIR). All characterization followed methodologies from our prior work,^19^ with additional details in the SI.

### Sorption and Desorption Experiments

For sorption experiments, stock solutions of target analytes (25 mM in methanol; 2.3–7.1 g/L) were stored at ∼5 °C and diluted 1000-fold with 1 mM potassium phosphate buffer (pH 7) to achieve 25 μM working concentrations. To initiate uptake experiments, these solutions were transferred into 40 mL amber glass vials, each containing 50 mg of the ENM composite material. Time-series data were collected by sacrificially sampling identically prepared vials at sequential time points (generally at 0.3, 0.6, 1, 2, 4, 8, 24, 36, and 48 h) to determine uptake kinetics and equilibrium behavior for each analyte-ENM combination. Experiments were conducted at room temperature in amber glass under both active mixing (rotated end-over-end for 48 h) and without mixing (static) conditions to evaluate the effect of mixing on the sorption rate and capacity.

Desorption or release experiments were performed with atrazine and metolachlor by first equilibrating identical ENMs in either “high” (1 mg/L) or “low” (5 μg/L) solution for 24 h. After equilibration, ENMs were transferred into separate 1 L bottles of deionized (DI) water, and desorption was monitored over 48 h through sacrificial sampling of both the aqueous-phase and methanol-extracted ENMs. At each time point, the DI water in the remaining reactors was replaced to simulate desorption into an infinite sink. Parallel experiments were conducted under active mixing (at 125 rpm on a New Brunswick Innova 2100 Orbital Shaker) and without mixing as well as under static conditions without water replacement to evaluate the effects of mixing and sink strength on release behavior. Sample processing followed established protocols, with additional details provided in Figure S1.

### Dynamic Micropollutant Uptake and Release Experiments

Using atrazine as a model pollutant because of its widespread use, frequent detection in Iowa's surface waters, and prior measurement at our field site, the uptake and release behavior of ENMs was evaluated under dynamic, stepwise changes in aqueous concentration to simulate fluctuating environmental conditions. Identical ENMs (10 mg each) were first placed in 300 mL of DI water without atrazine, then transferred every 2 h into fresh solutions with increasing atrazine concentrations (0.33 → 0.67 → 1 mg/L), followed by decreasing concentrations (1 → 0.67 → 0.33 mg/L), and finally into atrazine-free DI water, all while actively mixing at 125 rpm. At each step, an ENM was sacrificially sampled and analyzed by following previously described procedures. Atrazine concentrations above environmental relevance (up to 1 mg/L) were chosen for these studies to facilitate analysis of samples using HPLC with a diode array detector. Additional details are provided in Figure S2.

### Modeling of ENM Uptake, Release, and Step Dynamic Experiments

ENM uptake and accumulation were described using a one-compartment first-order kinetic model:^18,20^

1 $$C_{\mathrm{ENM}}=K_{\mathrm{ENM}‐W}\times C_{w}\times \left(1-e^{-\left(k_{e}\times t\right)}\right)$$

where *C*_ENM_ and *C*_w_ are the concentrations of the chemical in the ENM and water, respectively (mg/g and mg/L, respectively), *K*_ENM‑W_ is the equilibrium partitioning coefficient between the ENM and water (L/g), *k*_e_ is the elimination rate constant (1/d), and *t* is time (d). The model was also used to calculate the time required to reach 90% of equilibrium ( $t_{90\%}=\frac{\ln10}{k_{e}}$ ), which provides a convenient measure of the characteristic time scale for sorption equilibrium. For release experiments, a term for the fraction of the chemical that remained unreleased was included, consistent with biphasic release typically observed in sediment systems:^32,33^

2 $$\frac{C_{\mathrm{ENM}}}{C_{\mathrm{ENM}\left(t=0\right)}}=f+\left(1-f\right)\times e^{\left(-k_{e}\times t\right)}$$

where *f* represents the fraction of the chemical retained by the ENM. Step experiments were simulated using a dynamic mass-balance approach to account for uptake and partial retention:

$$C_{\mathrm{ENM}}\left(t\right)=C_{\mathrm{ENM}}\left(t-1\right)+\left(k_{u}\times C_{w}\left(t\right)-\left(1-f\right)\times k_{e}\times C_{\mathrm{ENM}}\left(t-1\right)\right)\times \Delta t$$ 3

where *k*_u_ (L/g/d) is the uptake rate constant, and Δ*t* is the time step. Time-series data for all experiments were fitted using nonlinear least-squares regression (Excel Solver) to estimate the relevant parameters (*k*_e_, *K*_ENM‑W_, *k*_u_, and *f*), and model performance was assessed using mean square error (MSE) and *R* ^2^ values. Fitted values of *f* and *K*_ENM‑W_ = *k*_u_/*k*_e_ were compared with equilibrium results from the uptake and release experiments to assess consistency across experimental designs. Parameter uncertainty was estimated using the jackknife resampling method.^18,34^

Overall, the model captured the experimental behavior well, with an average *R* ^2^ of 0.93 (±0.10) across all modeled systems. The exception was PAN with atrazine (*R* ^2^ = 0.39) due to an unusually high initial sorption. MSE values were low (≤0.07), and comparisons of fitted versus experimentally measured equilibrium parameters yielded *R* ^2^ values of 0.97–0.99. Dynamic experiments also showed good agreement for *f* (*R* ^2^ = 0.98), while differences in mixing conditions limited the direct comparison of kinetic parameters. Full modeling results, including all fitted parameters with uncertainties and system-specific comparisons, are provided in Tables S11 and S13 in the SI. Further methodological details are also provided in the SI.

### Determination of *K*_ENM‑W_ Values

To support field studies, we also determined equilibrium partition coefficients (*K*_ENM‑W_), defined as the ratio of the analyte concentration in the ENM (*C*_ENM_) to that in the aqueous phase (*C*_w_). Values of *K*_ENM‑W_ were used to back-calculate environmental aqueous concentrations based on the mass extracted from the ENMs and assuming equilibrium conditions during field deployment. These experiments were conducted in duplicate at three environmentally relevant concentrations (10, 100, and 500 μg/L) using a PAN/SDS_20_/COOH-CNT_10_ ENM composite. After equilibration, aqueous and methanol extracts were analyzed to quantify the analyte concentrations in both phases. *K*_ENM‑W_ values were then calculated by the linear regression of *C*_ENM_ versus *C*_w_, with the slope representing the partition coefficient. The *R* ^2^ and *p*-values were used to assess the fit quality and statistical significance of the regression models. Aqueous and solid-phase (ENM methanol extracts) samples were processed as previously described,^20^ with full analytical and procedural details provided in the SI.

### Field Deployments of ENM Samplers

Passive samplers fabricated using PAN/SDS_20_/COOH-CNT_10_ ENM composites were deployed in Muddy Creek (North Liberty, Iowa) in July 2022, within a watershed influenced by agricultural runoff (Figure S3). Each sampler consisted of a ∼10 × 10 cm ENM sheet enclosed between two 9.5 × 9.5 cm metal frames using self-locking zip ties, leaving an exposed area of 38.5 cm^2^ (Figure S4). Before deployment, samplers were soaked in methanol for at least 15 min to remove contaminants, air-dried, wrapped in Al foil, and stored at 5 °C.

At the field site, samplers were unwrapped and mounted in pairs on 1.5 m metal garden stakes using zip ties. Four pairs were deployed consecutively in two 2-h intervals; after the first deployment, samplers were retrieved and replaced with fresh pairs in the same locations (Figure S5). A 2 h interval was chosen because our laboratory uptake experiments showed that the materials equilibrate rapidly, even without mixing, making this duration sufficient for field equilibration. Additional samplers were left wrapped during fieldwork and served as field blanks to assess contamination and determine the limit of quantification. For comparison, grab samples (1 L) of water were collected at the start, midpoint, and end of the deployment period.

After retrieval, samplers and blanks were returned to the laboratory for extraction. ENMs were removed from the frames and placed in 10 mL glass vials containing a known volume of LC–MS grade methanol. Vials were rotated end-over-end for 24 h, and 1 mL aliquots of the extract were collected and stored in amber autosampler vials at 5 °C until analysis. Reported field aqueous concentrations of atrazine and metolachlor were calculated from ENM sampler results and experimentally derived *K*_ENM‑W_ values, where error bars of reported concentrations were calculated using the upper and lower bounds (±one standard deviation) of experimental *K*_ENM‑W_ values.

### Analytical Methods

Aqueous samples and methanol extracts of ENMs were analyzed via either high-performance liquid chromatography with a diode array detector (LC-DAD, Agilent 1100 series HPLC) or by using liquid chromatography with tandem mass spectrometry (LC–MS/MS Agilent 1360 Infinity LC with Agilent 6460 MS/MS). LC-DAD and LC–MS/MS methods were adapted from previously published methods and are summarized in the SI (Tables S5–S8).

### Quality Assurance and Quality Control

Laboratory blanks consisting of DI water, methanol, and ENMs stored in the laboratory were analyzed in parallel with experimental and field samples to rule out any laboratory contamination. Most sorption and desorption experiments were conducted in at least duplicate; corresponding uncertainties (shown as error bars) represent one standard deviation of replicate experiments. Due to limitations in the availability of specific ENM formulations, some experiments were conducted only once for certain materials or chemical targets. This approach was necessary given the time-intensive process of electrospinning sufficient quantities of ENM mass for sorption and desorption studies using a sacrificial sampling design. In these limited cases, displayed error bars represent reasonable estimates of uncertainty across all experimental uptake and release data. Field blanks were also analyzed alongside field samples to evaluate potential contamination during sample preparation and transport to the field site. Analysis of samples using LC–MS/MS in multiple reaction monitoring (MRM) mode involved assessing the ratio of quantitative and qualitative ions between calibration standards and field samples to confirm analyte presence. The limit of quantification (LOQ) was determined as the upper limit of the 95% confidence interval for atrazine and metolachlor from the field blanks (Table S9). Any ENM samplers deployed in the field that yielded atrazine or metolachlor concentrations at or below the LOQ were considered nonreportable.

## Results and Discussion

### ENM Characterization

As further detailed in the SI, SEM and BET analyses showed that surfactant inclusion generally had a limited impact on PAN/CNT_20_ dimensions, surface area, and pore volume except for a few notable instances (Table S10). Briefly, the addition of TBAB, which ATR-FTIR confirmed was retained in the PAN fibers (Figure S6), did not significantly change the average PAN/CNT_20_ diameter [e.g., 210 (±50) nm for PAN/NF-CNT_20_ and 240 (±110) nm for PAN/TBAB_20_/NF-CNT_20_; see SEM images and histograms of nanofiber diameters in Figure S7], but it did widen the distribution, likely through dispersing CNT aggregates, which may affect fiber uniformity. Similarly, for PAN/SDS_20_/CNT_20_ composites, average fiber diameters were slightly larger than unmodified PAN/CNT_20_ but not statistically significant due to a much broader size distribution [e.g., 200 (±40) nm for PAN/COOH-CNT_20_ and 310 (±140) nm for PAN/SDS_20_/COOH-CNT_20_]. Importantly, SDS-leached materials showed significantly higher specific surface areas, about 33 (±3.0) m^2^/g for leached COOH-CNT composites compared to 12 (±0.5) m^2^/g for pure PAN, nearly triple the surface area (p = 0.0001). This increase correlates with pore volume gains, which more than doubled from 37 (±8.0) and 43 (±6.0) μL/g in NF- and COOH-CNT composites, respectively, to 66 (±7.7) and 103 (±12) μL/g, after ENM washing to remove SDS.

### Influence of Quaternary Ammonium Surfactants on Uptake of Anionic Organic Pollutants

Figure 1a shows the influence of TBAB (at 20 wt %) in PAN and PAN/CNT_20_ composite ENMs on the uptake of 2,4-D, which is an anion at the pH value investigated (pH 7; 2,4-D p*K*_a_ of 2.98).^35^ We observed minimal uptake of 2,4-D with PAN, PAN/NF-CNT_20_, and PAN/COOH-CNT_20_, with corresponding *K*_ENM‑W_ values of 0.015 ± 0.002, 0.029 ± 0.002, and 0.064 ± 0.005 L/g, respectively (Table S11). Upon the addition of TBAB, uptake of 2,4-D was increased by ∼45-fold at equilibrium, with *C*_ENM_ values approaching 4 mg/g and a corresponding *K*_ENM‑W_ of 0.65 ± 0.4 L/g. Equilibrium also was achieved relatively quickly with these materials; the mass of 2,4-D in TBAB-containing ENMs was essentially constant after 1 day, with modeled *t*_90%_ values not exceeding 0.54 ± 0.22 days (Table S11), even in static (i.e., without mixing) experimental systems.

**Figure 1:**
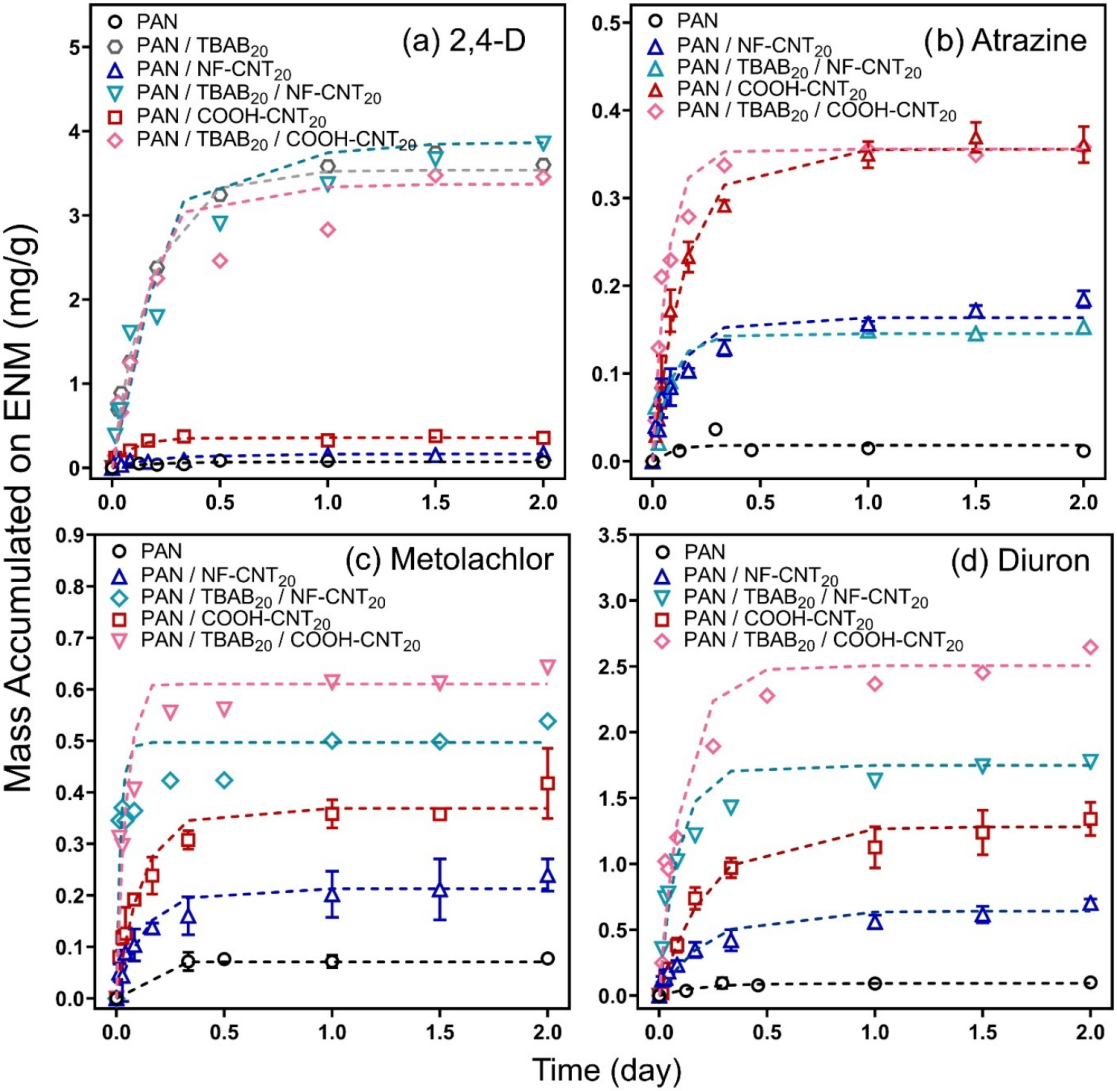
Effect of TBAB on uptake of (a) 2,4-D, (b) atrazine, (c) metolachlor, and (d) diuron by PAN, PAN/CNT_20_ (both NF- and COOH-CNTs), and PAN/TBAB_20_/CNT_20_. Initial aqueous conditions: 25 μM, pH 7, and without mixing conditions. Dashed lines correspond to the model described by eq 1.

For the uptake of anionic 2,4-D, we found a clear, beneficial effect upon including cationic TBAB in PAN and PAN/CNT_20_ ENMs. TBAB is a surface-segregating surfactant within polymer matrices,^36,37^ and quaternary ammonium groups are often used as strong base ion exchange sites.^36–40^ Indeed, we have previously demonstrated the use of TBAB-modified PAN in ion exchange by measuring the corresponding release of bromide ions during uptake of anionic chromate.^28^ Taken together, we presume that positively charged quaternary ammonium groups at or near the surface of PAN and PAN/CNT_20_ nanofibers function as electrostatic binding sites for anionic 2,4-D, presumably via a mechanism analogous to ion exchange. Indeed, the addition of TBAB resulted in some of the highest *K*_ENM‑W_ values, both experimentally and modeled (Table S11).

A concern we have previously identified with some surfactant-modified nanofibers is surfactant loss from the polymer over time.^28^ Accordingly, we extensively washed the TBAB-modified materials with DI water in an attempt to leach TBAB from the ENM prior to 2,4-D sorption experiments. The performances of PAN/TBAB_20_ and PAN/TBAB_20_/CNT_20_ remained the same after extensive washing, with identical 2,4-D uptake curves. As in our earlier work,^28^ we attribute the stability of TBAB to entanglement between the polymer and its four butyl tails.

### Influence of Quaternary Ammonium Surfactants on the Uptake of Neutral Organic Pollutants

Figure 1b shows uptake curves for atrazine, a neutrally charged, moderately polar (log *K*_ow_ 2.61)^20^ herbicide commonly detected in agriculturally impacted surface waters.^9,41^ Pure PAN ENMs exhibited minimal atrazine uptake (*K*_ENM‑W_ = 0.003 ± 0.001 L/g),^20^ but sorption increased in PAN/CNT_20_ formulations. Among these, COOH-CNTs led to the highest uptake (*K*_ENM‑W_ = 0.066 ± 0.001 L/g), while NF-CNTs showed an intermediate uptake (*K*_ENM‑W_ = 0.027 ± 0.001 L/g). Notably, the inclusion of TBAB into PAN/CNT_20_ ENMs had no discernible influence on the equilibrium uptake capacity for atrazine (identical *K*_ENM‑W_ values), although a consistent, albeit slight, increase in the rate of atrazine sorption was observed for TBAB-modified materials compared to PAN/CNT_20_ (Table S11).

We also conducted uptake experiments with two other common, neutrally charged and moderately polar herbicides, diuron (log *K*_ow_ 2.68; Figure 1d) and metolachlor (log *K*_ow_ 3.13; Figure 1c).^35^ For both diuron and metolachlor, the performance trend for materials without TBAB matched that observed for atrazine; unmodified PAN exhibited little uptake (*K*_ENM‑W_ = 0.015 ± 0.002 and 0.0106 ± 0.0004 L/g for diuron and metolachlor, respectively), whereas PAN/COOH-CNT_20_ exceeded the performance of PAN/NF-CNT_20_ by ∼2-fold. However, the addition of TBAB resulted in more uptake on each PAN/CNT_20_ composite, with PAN/TBAB_20_/COOH-CNT_20_ achieving the greatest degree of diuron and metolachlor uptake (*K*_ENM‑W_ = 0.43 ± 0.02 and 0.087 ± 0.002 L/g for diuron and metolachlor, respectively). Of the four chemical targets investigated, 2,4-D exhibited the highest partitioning (∼4 mg/g, *K*_ENM‑W_ = 0.66 ± 0.02 L/g on PAN/TBAB_20_, with or without NF-CNT), followed by diuron (∼3 mg/g, *K*_ENM‑W_ = 0.43 ± 0.02 L/g on PAN/TBAB_20_/COOH-CNT_20_), metolachlor (∼0.6 mg/g, *K*_ENM‑W_ = 0.087 ± 0.002 L/g on PAN/TBAB_20_/CNT_20_), and atrazine (∼0.4 mg/g, *K*_ENM‑W_ = 0.066 ± 0.002 L/g on PAN/COOH-CNT_20_) (Table S11).

In our prior investigation of neonicotinoid sorption on TBAB-containing ENMs, we observed a near-additive influence of TBAB and CNT wt % on neonicotinoid uptake when incorporated into PAN-based ENMs.^19^ Consequently, we proposed that neonicotinoid uptake occurs through a combination of hydrophobic (onto graphitic CNT surfaces) and more specific (via quaternary ammonium surface groups) binding interactions. Herein, we presume that similar factors are at play for the improved uptake of diuron and metolachlor on TBAB-containing ENMs. Atrazine, diuron, and metolachlor all share common functional groups (e.g., chlorine substituents, amine centers, and aromatic π-bonds), but a key distinction for diuron and metolachlor is the presence of a ketone group, where the associated oxygen possesses free lone pair electrons. The partial negative charge on this oxygen may help promote some degree of favorable interactions with the positively charged quaternary ammonium groups on the ENM surface. Additional work is needed to explore a broader range of neutral organic chemical targets and identify structural elements likely to benefit (e.g., electron rich) or hinder (e.g., electron deficient) their interaction with positively charged quaternary ammonium sites on these surfactant-modified ENM surfaces.

### Use of SDS as a Porogen to Improve PAN-ENM Performance

Figure 2 shows uptake curves for atrazine, diuron, and metolachlor using ENMs for which SDS was initially blended (at 20 wt % relative to PAN) and subsequently removed via extensive washing. For all analytes, uptake on SDS-treated ENMs was greater than on the corresponding unmodified ENM, ranging from 8- to 40-fold increases. For example, PAN/SDS_20_/NF-CNT_20_ (∼0.3 mg/g, *K*_ENM‑W_ = 0.051 ± 0.003 L/g) exhibited a ∼2-fold increase in atrazine uptake relative to PAN/NF-CNT_20_ (∼0.15 mg/g, *K*_ENM‑W_ = 0.032 ± 0.003 L/g) (Table S11). Increases of similar magnitudes were also observed for diuron and metolachlor. Another noteworthy observation was the relatively fast rate of chemical uptake into SDS-treated ENMs, with equilibrium generally achieved by 0.5 days and modeled *t*_90%_ values not exceeding 0.37 ± 0.12 days (Table S11), even in static (i.e., without mixing) experimental systems.

**Figure 2:**
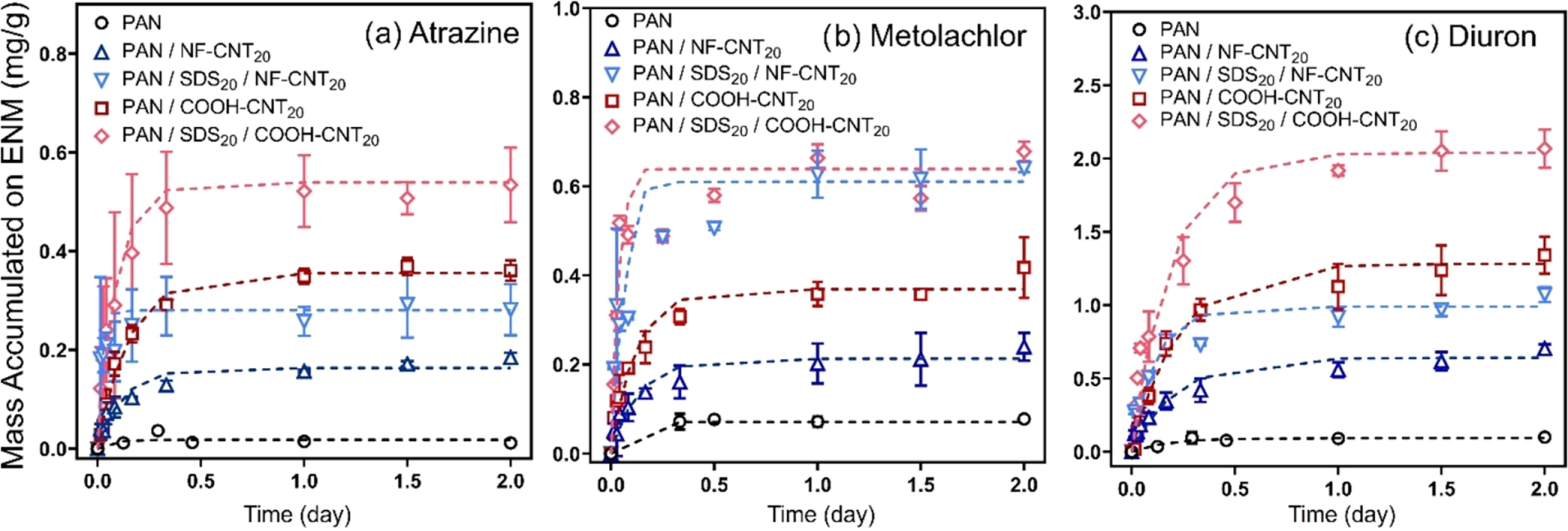
Effect of SDS on uptake of (a) atrazine, (b) metolachlor, and (c) diuron by PAN, PAN/CNT_20_ (both NF- and COOH-CNTs), and PAN/SDS_20_/CNT_20_ composites. Initial aqueous conditions: 25 μM (5.4 mg/L for atrazine, 7.1 mg/L for metolachlor, and 5.8 mg/L for diuron), pH 7, and without mixing conditions. Dashed lines correspond to the model described by eq 1.

These increases in chemical uptake with SDS-treated ENMs are on the order of the measured increases in the pore volume and surface area resulting from the use of SDS as a porogen (Table S10). Thus, improvements in chemical uptake are likely influenced by the greater surface area of SDS-treated ENMs. Moreover, we see no clear evidence that any residually bound SDS significantly alters ENM performance; for example, PAN/COOH-CNT_20_ generally outperformed PAN/NF-CNT_20_ by comparable amounts both with and without SDS (∼2-fold), except for metolachlor, for which the uptakes were similar.

### Desorption Studies to Determine Analyte Release from Surfactant-Modified ENMs

Results of desorption experiments with atrazine are shown in Figure 3a, which illustrates the change in normalized atrazine concentration within the ENM over time. Atrazine concentrations in Figure 3a were normalized to the initial amount of atrazine sorbed to the ENM at equilibrium (∼0.4 mg/g, depending on the ENM formulation). Desorption studies were conducted only with COOH-CNT-containing ENMs due to their higher sorption capacity relative to NF-CNT composites.

**Figure 3:**
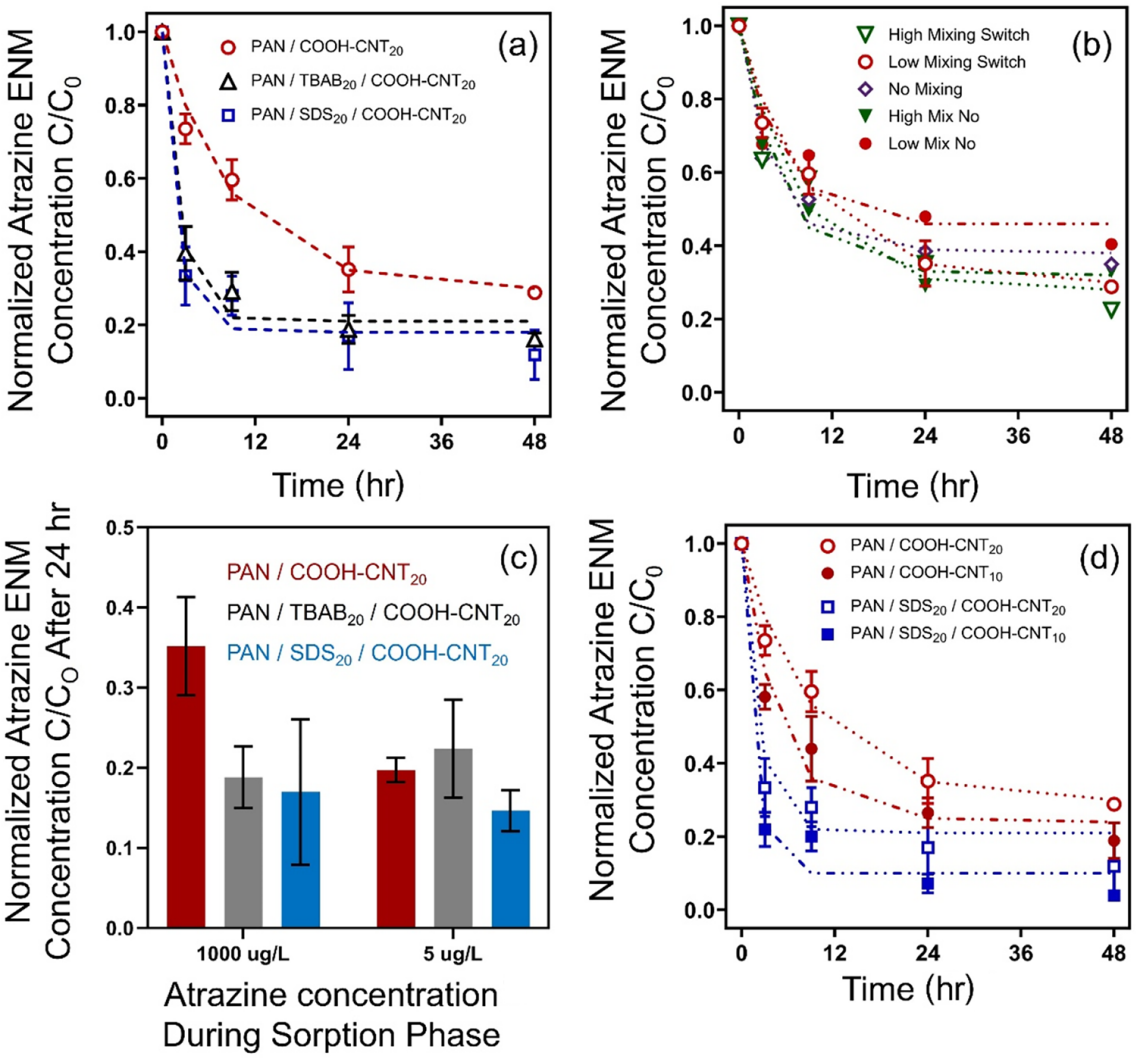
Desorption behavior of ENMs pre-equilibrated with atrazine (sorption conditions: ∼10 mg of ENM in 300 mL of 1 mg/L atrazine solution, pH 7 potassium phosphate buffer, 24 h). (a) Desorption behavior for PAN/COOH-CNT_20_, PAN/TBAB_20_/COOH-CNT_20_, and PAN/SDS_20_/COOH-CNT_20_ under low (50 rpm) mixing conditions. (b) Desorption behavior of PAN/COOH-CNT_20_ under different mixing conditions (reactors placed on orbital shaker at high 125 rpm, low 50 rpm, and without mixing) and with and without the exchange of clean water at each sampling point. (c) Comparison of normalized atrazine concentration remaining in PAN/COOH-CNT_20_, PAN/TBAB_20_/COOH-CNT_20_, and PAN/SDS_20_/COOH-CNT_20_ after 24 h when ENMs were pre-equilibrated in either a 1 mg/L or 5 μg/L atrazine solution, under low mixing conditions. (d) Desorption behavior of PAN/COOH-CNT formulations with and without SDS and either 20 or 10 wt % of COOH-CNT, under low (50 rpm) mixing conditions. Dashed lines correspond to the model described by eq 2.

Across all PAN/COOH-CNT formulations, the atrazine content within the ENMs decreased over time, consistent with desorption into the surrounding water under essentially infinite sink conditions, as atrazine was continuously removed through exchanges with clean water at each sampling point. The time scales of release were on the order of those previously observed for atrazine uptake (compare Figures 1b and 3a), with sorption equilibrium typically achieved within ∼1 day (average *t*_90%_ ≈ 0.5 days, Table S12) and the majority of atrazine desorption occurring over a comparable period of ∼1 day. Notably, elimination rates were independent of mixing conditions (Figure 3b), with a value of 3.8 ± 2.9 per day for PAN/COOH-CNT_20_ (Table S12), and atrazine concentration profiles were essentially identical in systems with and without mixing. This behavior suggests that the rate of atrazine release is not limited by transport across the aqueous surface film as it moves into the bulk solution but rather transport within the ENM material itself. Moreover, the release of atrazine did not appear to be influenced by its accumulation in the aqueous phase (Figure 3b) because experiments conducted without the exchange of clean water produced identical desorption profiles to those with clean water exchange.

The rate and extent of atrazine release did vary across the different ENM formulations (Figure 3a and Table S12). PAN/COOH-CNT_20_ only released ∼70% of the initially bound atrazine over 48 h (with ∼60% occurring within 24 h). In contrast, PAN/SDS_20_/COOH-CNT_20_ released ∼85% of the initially bound atrazine after 48 h, with most (∼70%) within 3 h. Similar behavior was observed for PAN/TBAB_20_/COOH-CNT_20_. Additional experiments with these formulations also revealed that the rate and extent of atrazine release were independent of the initial mass of atrazine bound to the ENM, particularly for TBAB-modified and SDS-treated materials (Figure 3c).

The faster rate and greater extent of release from surfactant-modified ENMs are notable. For example, the inclusion of TBAB increased the elimination rates by ∼3-fold and the total amount released from ∼70 to 80% compared with the unmodified PAN/COOH-CNT_20_ composites (Figure 3b and Table S12). The observation of different atrazine desorption behavior for these same ENMs suggests that while TBAB integration does not promote atrazine uptake, the positive charge on the quaternary ammonium group may result in weaker specific binding interactions such that bound atrazine is more susceptible to release. Similarly, one might anticipate that the introduction of pore space using SDS as a porogen would produce slower elimination rates due to potential mass transfer limitations. However, no such limitations were observed in these higher porosity materials, where the elimination rate with SDS was very similar to that of TBAB (*k*_e_ = 10 ± 2.9 vs 11.7 ± 7.3 per day, Table S12).

Also evident for the surfactant-modified ENM were two distinct phases of desorption. An initial, short phase of rapid atrazine release, behavior consistent with a first-order desorption model commonly applied to passive sampler performance,^18,42^ was followed by a longer second phase in which the elimination rate was much slower, resembling the gradual chemical release observed in sediment systems,^32,33^ as described by eq 2. We theorize that this desorption profile is indicative of at least two distinct types of sorption sites within the ENMs. This could be related to some atrazine binding sites being more accessible than others (e.g., surface versus bulk or internal pore sites). Alternatively, it could indicate sites with different mechanisms of atrazine binding; for example, atrazine uptake on the surfactant-modified PAN driven by weaker, specific binding interactions would likely result in faster rates of release than stronger binding driven by nonspecific, hydrophobic partitioning on the embedded CNTs.

Finally, because 15–20% of the initially bound atrazine remained associated with TBAB-modified and SDS-treated PAN/CNT composites after 48 h (Figure 3a), we also explored how atrazine desorption changes for ENMs with a lower CNT content (Figure 3d). Although ENMs with 10 wt % CNT-COOH exhibited a lower degree of atrazine sorption [e.g., the *K*_ENM‑W_ of atrazine decreased from 0.097 ± 0.002 L/g with PAN/SDS_20_/COOH-CNT_20_ (Table S11) to 0.052 ± 0.006 L/g with PAN/SDS_20_/COOH-CNT_10_ (Figure S8b)], we observed a greater rate and extent of release at 10 wt % COOH-CNT loading. In fact, for PAN/SDS_20_/COOH-CNT_10_ nearly 96 (±0.5) % of the initially bound atrazine was released into solution after 48 h, with the majority (∼80%) occurring within 3 h (Table S12).

The observation of greater rates and extents of release at lower embedded COOH-CNT concentrations holds both practical and fundamental significance. Practically, in developing ENMs for use as fast equilibrium passive samplers, the COOH-CNT concentration will need to balance sorption capacities (that increase with increasing COOH-CNT wt %) with sorption reversibility (that increases with decreasing COOH-CNT wt %). More fundamentally, this result suggests that more irreversible atrazine binding likely occurs on the surface of the embedded COOH-CNTs, whereas more reversible sorption sites are presumably linked to the polymer-modified surfactant.

### ENM Response to Temporal Changes in Atrazine Concentration

Figure 4 shows the corresponding uptake and release curves generated for four different ENM formulations in response to increasing (from 0 to 1 mg/L) and then decreasing (from 1 to 0 mg/L) solution phase atrazine concentrations. Data are shown for the mass of atrazine measured within the ENM over time in response to these changes in the solution phase concentration.

**Figure 4:**
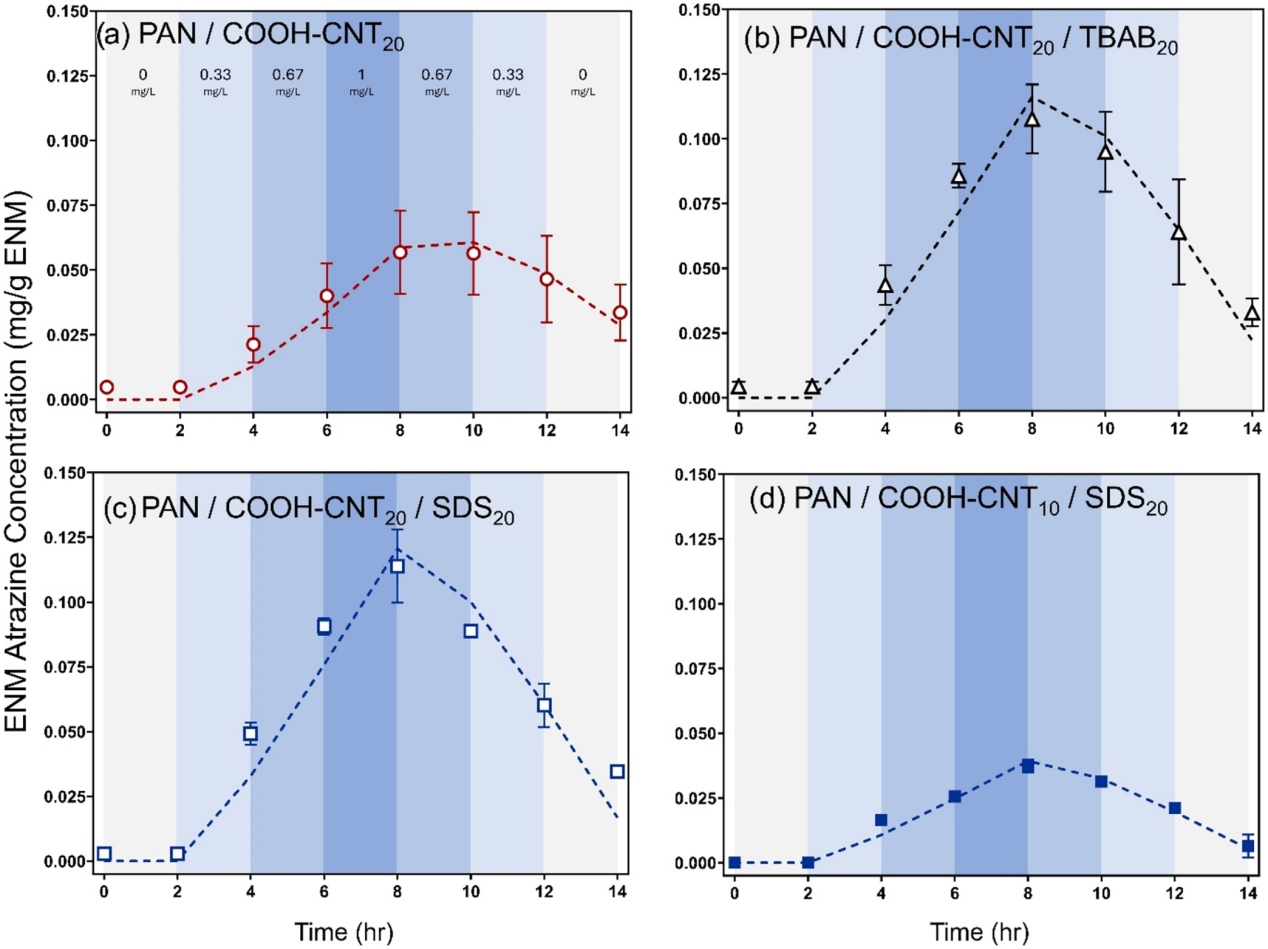
Atrazine ENM concentration over time in response to sequential changes in aqueous atrazine concentration for various ENM formulations as indicated. ENMs were first placed in clean DI water at time 0 h. After 2 h, and every 2 h thereafter, the ENMs were placed in a new solution with an increasing aqueous atrazine concentration up to 1 mg/L atrazine, followed by decreasing aqueous atrazine concentration until finally the ENMs were placed into clean DI water again. The experiment was performed with ∼10 mg of ENM placed into 300 mL of solution under high (125 rpm) mixing conditions. Dashed lines correspond to the model described by eq 3.

There are several notable features in the uptake and release curves in Figure 4. First, the surfactant-modified ENMs exhibited greater uptake relative to corresponding ENMs without surfactant (∼100 vs ∼50 mg/g). This reflects the greater rates of atrazine sorption on surfactant-modified ENMs, where the shorter time intervals of these experiments (i.e., 2 h exposure to each aqueous atrazine concentration) relative to equilibrium studies (Figures 1 and 2) result in more pronounced differences in uptake on the surfactant-modified ENMs. Second, while PAN/SDS_20_/COOH-CNT_20_ and PAN/TBAB_20_/COOH-CNT_20_ exhibited nearly identical kinetic profiles, their atrazine concentration profiles are not consistent with fully reversible uptake and release, as the mass of atrazine remaining in the ENMs at the end of the 14 h experiment was not zero (i.e., not the same as the start of the experiment). This is likely attributable to the fraction of irreversibly bound atrazine on the 20 wt % COOH-CNT ENMs, as observed in desorption studies (Figure 3a).

Most promising for application in a fast equilibrium passive sampling device is PAN/SDS_20_/COOH-CNT_10_. For this ENM, the atrazine concentration profile followed the expected trend for a reversible sorption process, with the bound atrazine concentration nearly returning to zero at the conclusion of the 14 h experiment. Further, the concentration of ENM-bound atrazine was roughly equivalent at the end of each interval with an identical aqueous-phase atrazine concentration, regardless of whether these intervals occurred during the period of increasing or decreasing aqueous-phase atrazine concentration. For example, after 2 h of exposure to 0.67 mg/L (after an increase from 0.33 mg/L), the ENM-bound atrazine concentration was 25 mg/g, a value nearly identical to the ENM-bound atrazine concentration measured after 2 h of exposure to 0.67 mg/L (after a decrease from 1 mg/L).

Finally, the concentration profiles in Figure 4 illustrate the trade-offs associated with achieving more reversible atrazine sorption at a lower CNT concentration of 10 wt %. Although able to achieve nearly reversible atrazine uptake and release, the extent of atrazine sorption on PAN/SDS_20_/COOH-CNT_10_ is roughly one-half of that for PAN/SDS_20_/COOH-CNT_20_. For equilibrium passive sampling applications, a higher uptake capacity is preferred to improve limits of detection (i.e., more target analyte mass could be recovered from sampler extraction). Overcoming the lower inherent capacity of PAN/SDS_20_/COOH-CNT_10_ will likely require a larger total mass of ENM to be deployed in the field to increase the total amount of target analyte captured during a sampling event. Finaly, we note that in all cases the kinetic model fit the experimental observations well, and the parameters obtained from these fits were consistent with those derived from the uptake and release experiments (Table S13).

### Laboratory Studies in Support of ENM Field Deployment

To support ENM deployment at Muddy Creek, North Liberty, IA, for measurement of atrazine and metolachlor, additional laboratory studies were first conducted with the optimal ENM formulation, PAN/SDS_20_/COOH-CNT_10_. These laboratory experiments were intended to validate the reversibility of metolachlor sorption and determine *K*_ENM‑W_ values for atrazine and metolachlor at environmentally relevant concentrations. These *K*_ENM‑W_ values would then be used to determine aqueous-phase concentrations based on the amount of each analyte extracted from the ENM after field deployment, assuming that the sampler had reached equilibrium with the aqueous phase.

Results from desorption experiments with PAN/SDS_20_/COOH-CNT_10_ that were previously equilibrated with 1 mg/L metolachlor are shown in Figure S8a, along with the corresponding data for atrazine (from Figure 3d) for comparison. Desorption of metolachlor occurred at a rate comparable to that of atrazine, with ∼80% of the total metolachlor mass released within 3 h. Thereafter, the rate of metolachlor release considerably slowed, and essentially no more desorption was observed after 24 h. Ultimately, 8.0 ± 0.4% of metolachlor mass remained bound to the ENM after 48 h, resulting in a small but reproducible difference in release behavior compared with atrazine (6.0 ± 2.3%). We attribute this to the greater hydrophobicity of metolachlor, which possesses at log *K*_ow_ value ∼0.5 log units larger than atrazine (Table S3). We anticipate that sorption on COOH-CNTs would be stronger for more hydrophobic chemicals, potentially explaining the lesser degree of metolachlor release even at a lower concentration of 10 wt %.

Figure S8b shows sorption isotherms for atrazine and metolachlor on PAN/SDS_20_/COOH-CNT_10_ that were collected at concentrations (i.e., 10–500 μg/L) representative of what might be encountered at Muddy Creek based on historical monitoring of Iowa surface waters.^43,44^ Experimentally measured (via extraction) ENM concentrations for atrazine and metolachlor varied linearly with the equilibrium aqueous-phase concentrations. Accordingly, values of *K*_ENM‑W_ were calculated from linear regression analysis and determined to be 0.052 (±0.006) and 0.034 (±0.004) L/g for atrazine and metolachlor, respectively (*R* ^2^ of 0.86 and 0.95; *p* = 0.0005 and <0.0001, respectively). These values agree reasonably well with estimated *K*_ENM‑W_ values determined from single-point measurements after equilibration of the same ENM formulation with 1 mg/L atrazine (0.065 ± 0.017) and metolachlor (0.056 ± 0.011) for use in desorption studies (Figure 4a). The relatively good agreement in *K*_ENM‑W_ values across a broad concentration range (from 10 μg/L to 1 mg/L) suggests that there is an excess of sorption sites in this ENM formulation, indicating a partitioning process and making it suitable for use across a wide range of dissolved atrazine and metolachlor concentrations in environmental systems.

### Field Deployments in Muddy Creek, North Liberty, IA

Results from the analysis of ENM samplers deployed at Muddy Creek (North Liberty, IA) are shown in Figure 5 along with the results of the corresponding grab sample analysis. Aqueous concentration data for atrazine and metolachlor are provided for each of the four sampling locations, and data are shown as a function of time across the two consecutive, 2 h deployments. The panels of the figure are oriented corresponding to how ENM samplers were oriented in the creek, with flow from west to east.

**Figure 5:**
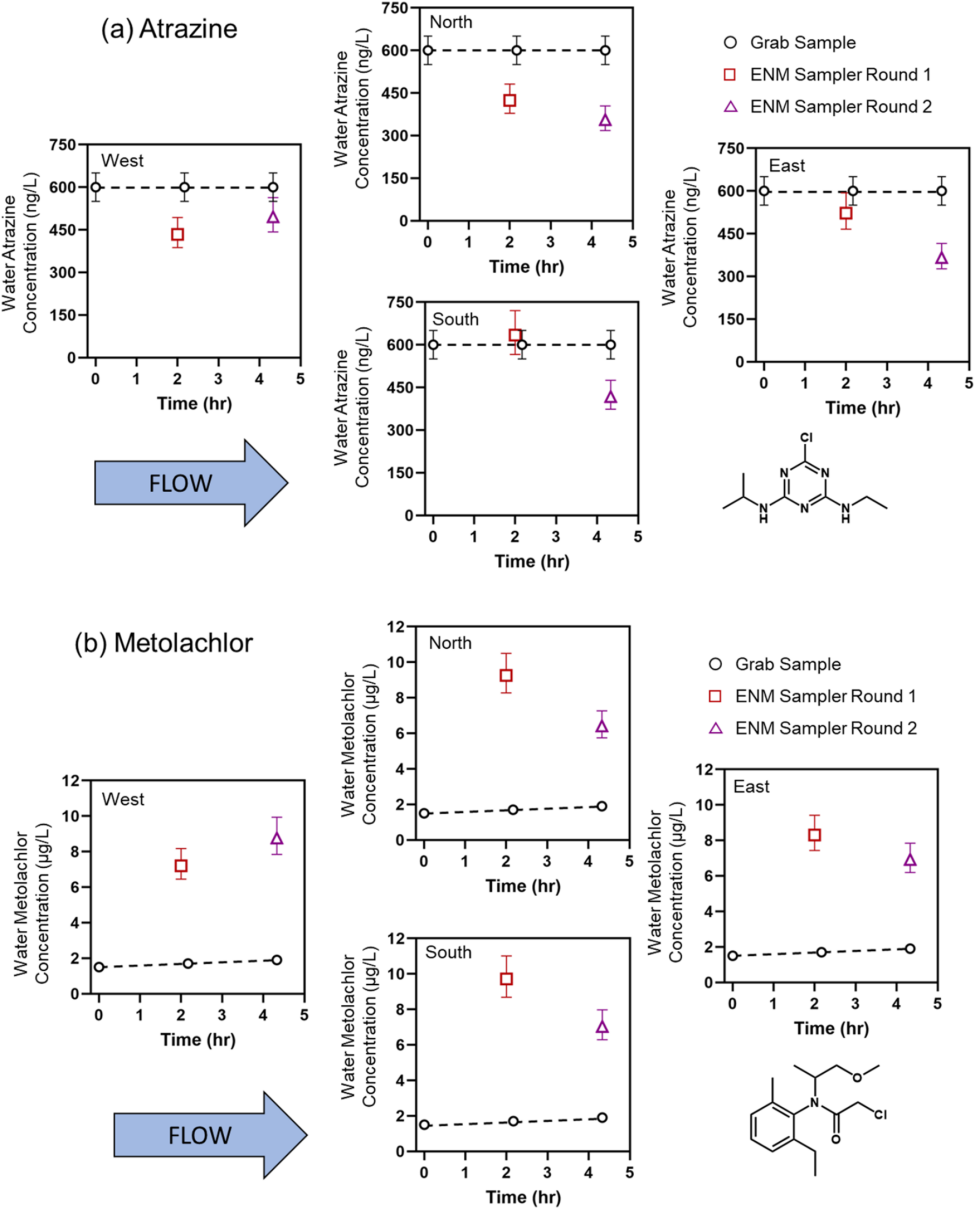
Aqueous concentrations for (a) atrazine and (b) metolachlor determined from ENM samplers and grab sample analysis at Muddy Creek. Results of three different grab samples collected over the ENM deployment period are shown in black (error bars represent an uncertainty of ±50 ng/L as reported by the analytical laboratory), while results from the first and second 2 h deployment of ENM samplers are represented in red and purple, respectively. As fast equilibrium passive samplers, calculated atrazine and metolachlor concentrations from the ENM samplers most accurately represent aqueous contaminant concentration at the end of their 2 h deployment, and the concentrations are plotted as such. Each one of the four plots (West, North, South, East) represents a single stake with two attached ENM samplers (Figure S5), and the extracts of both were combined during analysis to increase the total analyte mass available for detection and quantification.

Atrazine was detected in all ENM and grab samples. Analysis of aqueous grab samples revealed a constant atrazine concentration of 0.60 (±0.05) μg/L over the entire ∼4 h duration of the ENM deployment. Calculated aqueous concentrations based on ENM sampler concentrations and laboratory-measured *K*_ENM‑W_ values ranged from 0.35 to 0.63 μg/L, averaging 0.50 (±0.10) μg/L during the first 2 h deployment and 0.41(±0.06) μg/L during the second 2 h deployment (Figure 5a). Field blank ENM concentrations were generally less than one-third of the deployed ENM concentrations, and all ENM samplers were sufficiently above the LOQ (Tables S9 and S14). The level of agreement between ENM and grab sample analysis is quite reasonable and is consistent with our prior measurement of atrazine levels in Muddy Creek when using PAN/CNT composites without SDS modification.^20^

Metolachlor was also detected in all ENM and grab samples from Muddy Creek (Figure 5b). Unlike atrazine, there is a clear and relatively large divergence between the results obtained by grab samples and those from ENM samplers. Grab sample analysis detected metolachlor concentrations increasing from 1.5 (±0.1) to 1.9 (±0.1) μg/L over the course of the ∼4 h deployment. Calculated aqueous metolachlor concentrations from ENM samples and laboratory-measured *K*_ENM‑W_ values were anywhere between 3.3 and 6.5 times higher, ranging from 6.4 μg/L to 9.7 μg/L and averaging 8.62 μg/L ± 1.11 during the first 2 h deployment and 7.28 μg/L ± 1.02 during the second 2 h deployment (Table S15).

The contrasting performance of the ENM sampler toward atrazine and metolachlor is not entirely understood. One potential explanation could relate to the greater hydrophobicity of metolachlor (log *K*_ow_ 3.13) relative to atrazine (log *K*_ow_ 2.6), which may impact the reversibility of metolachlor sorption on CNT-containing formulations. Although desorption studies (Figure S8a) showed relatively modest differences in the extent of atrazine and metolachlor release over 48 h, we note that these desorption trials were conducted after exposing the PAN/SDS_20_/COOH-CNT_10_ ENM to 1 mg/L of each pesticide, producing 70 μg/g of initially bound atrazine and 50 μg/g of initially bound metolachlor at the start of the release experiment. These conditions are well above the concentrations to which the passive sampler was exposed in Muddy Creek (e.g., for atrazine and metolachlor, grab concentrations were ∼1600-fold and 500-fold less, respectively). If there is a larger reservoir of irreversible sorption sites in PAN/SDS_20_/COOH-CNT_10_ ENMs for metolachlor than atrazine, then we might anticipate that nonequilibrium (i.e., irreversible) partitioning could lead to the accumulation of excess metolachlor in the sampler over time. After extraction into methanol, the release of this accumulated, irreversibly bound metolachlor would to lead to artificially high estimates of aqueous metolachlor concentration, as we observed when assuming reversible equilibrium. To address this, additional studies are likely warranted probing the desorption of target chemicals from ENMs at lower, more environmentally relevant concentrations, and considering how physicochemical descriptors like log *K*_ow_ values correlate to the extent of sorption reversibility for these and other ENM-CNT composite formulations.

## Conclusions

This study demonstrates that surfactant modification of ENMs can significantly enhance their sorption capabilities for both charged and neutral contaminants commonly found in surface waters, including atrazine, metolachlor, diuron, and 2,4-D. The incorporation of TBAB introduces positively charged sites to the ENM surface, which improves the sorption of anionic 2,4-D through favorable electrostatic interactions. Interestingly, TBAB-modified ENMs also showed improved sorption for some neutral species, such as metolachlor and diuron, likely due to interactions with electron-rich functionalities on these molecules. However, the presence of TBAB did not influence the overall extent of atrazine uptake, highlighting the importance of the contaminant structure in determining sorption behavior.

Use of SDS increased the pore volume and surface area of the ENMs. This structural change led to a higher sorption capacity across all tested contaminants. Importantly, SDS-treated ENMs also exhibited enhanced sorption reversibility. Focusing on atrazine, we found that while some sorption to ENMs was irreversible (particularly due to the CNT content), surfactant-modified ENMs released a higher fraction of sorbed atrazine, indicating more reversible behavior. ENMs with lower CNT content generally exhibited greater reversibility, although at the cost of reduced sorption capacity. Additionally, these materials responded quickly to short-term changes in contaminant concentrations, aligning with the performance criteria for fast equilibrium passive samplers.

The findings herein suggest that surfactant-modified ENMs can be tailored to improve both contaminant uptake and sampling dynamics. TBAB-functionalized ENMs may be best suited for the rapid detection of specific charged species such as 2,4-D, even if their sorption mechanism is less reversible. On the other hand, SDS-treated ENMs offer a greater potential for passive sampling applications, given their enhanced capacity and reversibility.

Among the formulations tested, PAN/SDS_20_/COOH-CNT_10_ demonstrated nearly complete reversible uptake of atrazine and was selected for proof-of-concept field trials. For metolachlor, we found that although sorption was mostly reversible on PAN/SDS_20_/COOH-CNT_10_, a small irreversible bound fraction persisted, likely due to metolachlor’s higher hydrophobicity. Sorption isotherms conducted at environmentally relevant concentrations showed consistent equilibrium behavior for both atrazine and metolachlor, indicating a wide linear range of uptake.

Field trials using PAN/SDS_20_/COOH-CNT_10_ for fast passive sampling revealed promising but mixed results. For atrazine, ENM-derived concentrations aligned closely with grab samples after short 2 h deployments. In contrast, metolachlor concentrations were overestimated, likely due to its greater irreversible sorption. This highlights the need for future work to better understand how contaminant physicochemical properties, such as hydrophobicity, affect reversible binding at environmentally relevant concentrations and, in turn, sampler performance.

Despite the variability in field performance, this work underscores the promise of PAN/CNT composites—particularly those treated with SDS, for use as fast equilibrium passive samplers. These materials are compact, lightweight, and easy to deploy and allow the rapid collection of water quality data over short time periods. Their quick responsiveness makes them ideal for capturing transient events like runoff, and their ease of analyte recovery via methanol extraction makes them functionally similar to solid-phase extraction tools. More testing is certainly needed, including exploring performance toward other polar to moderately hydrophobic organics beyond the chemical targets included in this study. With further refinement, such ENMs could offer a valuable complement to traditional passive sampling technologies, especially where high-frequency or short-duration monitoring is needed.

## Supplementary Material



## Notes

Nonfunctionalized and carboxylated carbon nanotube (NF-CNT and COOH-CNT) characteristics provided by the vendor (Table S1); surfactant structures and select physiochemical properties of two surfactants (Table S2); the structure and select physiochemical properties of the target analytes investigated (Table S3); electrospinning parameters for different ENM formulations (Table S4); uptake ENM model (text); description of the partition coefficient determination (text); experiment setup and basic actions taken during the course of a sorption reversibility experiment (Figure S1); experiment setup and basic actions taken during the course of a sorption dynamic uptake and release experiment (Figure S2); satellite image of sampling location, Muddy Creek, North Liberty, IA (Figure S3); a close-up view of an ENM sampler (Figure S4); field deployment configuration of ENM samplers in Muddy Creek, North Liberty, IA (Figure S5); summary of LC-DAD parameters for target analytes (Table S5); summary of LC–MS/MS parameters. Methods adapted from USGS (Table S6); MS/MS parameters. Methods adapted from USGS (Table S7); MRM parameters. Methods adapted from USGS (Table S8); description of recoveries from ENM (Text); average field blank ENM concentration with standard deviation, and limit of quantification (Table S9); ATR-FTIR method (test); surface area, pore volume, and average nanofiber diameter of ENM (Table S10); ATR-FTIR PAN and PAN/TBAB spectra (Figure S6); representative SEM images of various ENM formulations and histograms of nanofibers within various ENM formulations (Figure S7); uptake ENM model results (text); summary of equilibrium partition coefficients and kinetic parameters from uptake experiments (Table S11); summary of kinetic parameters and fraction of atrazine retained by PAN from release experiments (Table S12); summary of kinetic parameters and fraction of atrazine retained by PAN from dynamic step experiments (Table S13); normalized analyte ENM concentration over time for PAN/SDS_20_/COOH-CNT_10_ and low concentration sorption isotherm for PAN/SDS_20_/COOH-CNT_10_ with atrazine and metolachlor (Figure S8); results from ENM and grab sample analysis from Muddy Creek, North Liberty, IA (Tables S14 and S15) (PDF)
