# Supplementary material for: Polymeric Electrospun Nanofiber Composites as Fast Equilibrium Passive Samplers: Integration of Surface Functionalities and Porosity to Improve Organic Chemical Uptake
Source: ACS Environ Au. 2025 Dec 11;6(2):225–37. doi: 10.1021/acsenvironau.5c00183 (PMC13003359; doi:10.1021/acsenvironau.5c00183)
Supplement: Supplementary file 1 [file vg5c00183_si_001.pdf]

Supporting Information for:

Polymeric Electrospun Nanofiber Composites as Fast Equilibrium Passive Samplers: Integration of Surface Functionalities and Porosity to Improve Organic Chemical Uptake

Matthew R. Nagorzanski,[a] *Jiajie Qian,*[a,b] *Sarah A. Crane,*[c] *David M. Cwiertny,*[a,b,c*] *and Andres Martinez,*[a]*

a – Department of Civil & Environmental Engineering, IIHR – Hydroscience & Engineering
b – Department of Chemical & Biochemical Engineering
c – Department of Chemistry
The University of Iowa, Iowa City, IA, USA, 52246

*Co-corresponding authors

DMC: Phone (319) 335-1401; Email david-cwiertny@uiowa.edu

AM: Phone (319) 471-3070; Email andres-martinez@uiowa.edu

Prepared for:
ACS Environmental Au

November 11, 2025

15 Tables, 8 Figures, 23 pages

Experimental

Reagents

Table S1. Non functionalized and carboxylated carbon nanotube (NF-CNT and COOH-CNT) characteristics provided by the vendor (Cheap Tubes, Inc.).

	NF-CNT	COOH-CNT
Surface Functionality	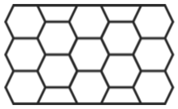	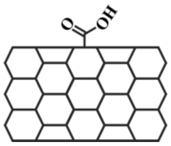
Wall Type	Multi-walled CNT	Multi-walled CNT
Outer Diameter	< 8 nm	< 8 nm
Length	0.5 - 2.0 µm	0.5 - 2.0 µm
Purity	> 95 wt%	> 95 wt%
Ash	< 1.5 wt%	< 1.5 wt%
Functional Content	-	3.86 wt.% (COOH groups)

Table S2. Surfactant structures and select physiochemical properties of two surfactants used herein.[1]

	TBAB	SDS
Structure	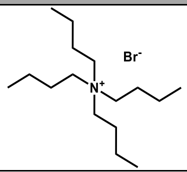	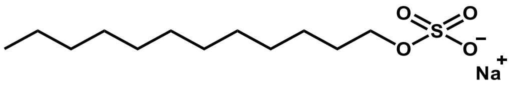
Molecular formula	$C_{16}H_{36}BrN$	$NaC_{12}H_{25}SO_4$
MW (g/mol)	322.37	288.37
Charge	Positive	Negative
Water Solubility (20 °C; g/L)	600	250
Solubility in DMF (20 °C)	Soluble	Soluble

Table S3. The structure and select physiochemical properties of the target analytes investigated herein.[2]

Chemical	Structure	MW (g/mol)	logK_{OW}	Water Solubility (25 °C; g/L)	Hydrogen bonds (count)	pK_a (25 C)	Charge @ pH ~7
Atrazine	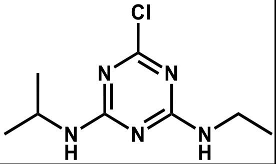	215.68	2.6	0.0007	H-donor (2)<br>H-acceptor (5)	2.3	Neutral
Diuron	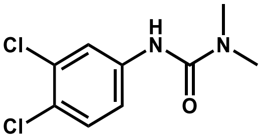	233.09	2.7	0.00042	H-donor (1)<br>H-acceptor (3)	14	Neutral
2,4-D	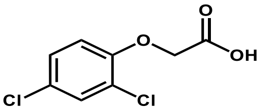	221.04	2.8	0.0054	H-donor (1)<br>H-acceptor (3)	2.98	Negative
Metolachlor	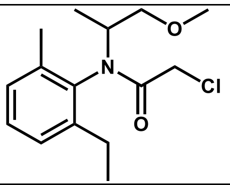	283.79	3.13	0.0053	H-donor (0)<br>H-acceptor (3)	1.45	Neutral

Electrospinning

Table S4. Electrospinning parameters for different ENM formulations.

	**Sol Gel Composition (relative to total sol gel mass)**			**Electrospinning Parameters**		
Mat Type (wt% of ingredients relative to PAN)	wt% PAN	wt% CNT	wt% Surfactant	Voltage Applied (kV)	Distance to Collector (cm)	Flow Rate (mL/hr)
PAN	8	N/A	N/A	15	10	0.5
PAN/$TBAB_{20}$	8	N/A	1.6	18	10	0.5
PAN/NF-CNT_{20}	8	1.6	N/A	16	10	0.5
PAN/COOH-CNT_{20}	8	1.6	N/A	16	10	0.5
PAN/$TBAB_{20}$/NF-CNT_{20}	8	1.6	1.6	18	10	0.5
PAN/$TBAB_{20}$/COOH-CNT_{20}	8	1.6	1.6	18	10	0.5
PAN/SDS_{20}/NF-CNT_{20}	8	1.6	1.6	18	10	0.5
PAN/SDS_{20}/COOH-CNT_{20}	8	1.6	1.6	18	10	0.5

Modeling of ENM Uptake, Release, and Step Dynamic Experiments

ENM uptake and accumulation were described using a one-compartment first-order kinetic model, as in our prior work (eq. S1): [3, 4]

$$C_{ENM} = K_{ENM-W} \times C_w \times \left(1 - e^{-(k_e \times t)}\right) \text{ (eq. S1)}$$

where C_{ENM} and C_w are the concentrations of the chemical in the ENM and water, respectively (mg/g and mg/L), $K_{ENM\text{-}W}$ is the equilibrium partitioning coefficient between the ENM and water (L/g), k_e is the elimination rate constant (1/d), and t is time (d). Time-series data for C_{ENM} and C_w (Figs. 1- 2) were fitted to eq 1 using nonlinear least-squares regressions in Excel (Solver tool) to estimate k_e and $K_{ENM\text{-}W}$. Solver minimized the sum of squared differences between observed and model-predicted C_{ENM}, defined as (eq. S2):

$$SSE = \sum_{i=1}^{n} (C_{ENM,obs,i} - C_{ENM,mod,i})^2 \text{ (eq. S2)}$$

where $C_{ENM,obs,i}$ and $C_{ENM,mod,i}$ are the observed and modeled ENM concentrations at time i, and n is the number of observations. Model performance was evaluated using the mean square error (MSE = SSE/n) and the coefficient of determination (R^2) between model predictions and observations C_{ENM}. The fitted $K_{ENM\text{-}W}$ values were compared with experimentally derived equilibrium partition coefficients (C_{ENM}/C_w) using R^2. The model was also used to calculate the time required to reach 90% of equilibrium ($t_{90\%}$) using the following equation, which provides a convenient measure of the characteristic timescale for sorption equilibrium (eq. S3).

$$t_{90\%} = \frac{\ln 10}{k_e} \text{ (eq. S3)}$$

For the release experiments (Fig. 3), a fraction of the chemical that does not release from the ENM within the experimental timescale was included, as preliminary data suggest that part of the mass remains unreleased, in line with previous observations of biphasic (fast and slow) chemical release reported for sediment systems.[5, 6] The release model is expressed as (eq. S4):

$$\frac{C_{ENM}}{C_{ENM\,(t=0)}} = f + (1 - f) \times e^{(-k_e \times t)} \text{ (eq. S4)}$$

where f represents the fraction of chemical retained by the ENM. Nonlinear least-squares regression (Excel Solver) was again used to fit k_e and f to the time-series data from $C_{ENM}/C_{ENM\,(t=0)}$, and model performance was assessed using the MSE and R^2 as described above. The fitted f values were compared with experimentally derived f (average of the last 2 measurements, Fig. 3) using R^2.

For the step experiments (Fig. 4), we modeled using a dynamic mass-balance approach to simulate the temporal evolution of the chemical concentration in the ENM phase (eq. S5):

$$C_{ENM}(t) = C_{ENM}(t-1) + (k_u \times C_w(t) - (1-f) \times k_e \times C_{ENM}(t-1)) \times \Delta t \text{ (eq. S5)}$$

where $C_{ENM}(t)$ and $C_w(t)$ are the ENM and water concentrations at time t, respectively, k_u (L/g/d) is the uptake rate constant, and Δt is the time step. The model was fitted to time-series data using nonlinear least-squares regression (Excel Solver), minimizing the sum of squared differences between observed and model-predicted values as described above (i.e., MSE and R^2). The parameters k_u, k_e, f and $K_{ENM\text{-}W}$ were fitted simultaneously, and the fitted values of f and $K_{ENM\text{-}W} = k_u/k_e$ were compared to the corresponding experimental results from Figs. 1 and 3 to assess model consistency. Parameter uncertainty was estimated using the jackknife resampling method.[3, 7]

Partition Coefficient Determination

ENM-water partition coefficient (i.e., $K_{\text{ENM-W}}$ values) for atrazine and metolachlor were also determined for use in aqueous phase concentration (C_w) calculations via eq. S6,

$$K_{ENM-W} = {C_{ENM}}/{C_W} \text{ (eq. S6)}$$

To determine log$K_{\text{ENM-w}}$ values, equilibrium sorption experiments were conducted with atrazine and metolachlor on PAN/SDS_{20}/COOH-CNT_{10}. Notably, these sorption experiments were performed in duplicate at three concentrations, 10, 100, and 500 µg/L, that were far lower than previous uptake experiments with comparable materials [20] to better simulate environmentally relevant concentrations. Pieces of ENM composite material (~2 cm × 2 cm, ~10 mg) were placed in aqueous solutions of either atrazine or metolachlor diluted from methanol based stock solutions (3.1 and 1.9 g/L respectively) to achieve a final ENM mass loading of 1.25 g/L. ENM-containing solutions were then rotated end-over-end until achieving sorption equilibrium (i.e., no significant additional changes in concentration over time) after 1 day. Next, a 1 mL aqueous sample was transferred into a 2 mL amber glass autosampler vial and stored in a 5 °C fridge until analysis to determine the aqueous phase concentration of herbicide. ENMs were then placed in a pre-measured volume of methanol within a 10 mL clear glass vial and rotated

end-over-end to allow any sorbed atrazine or metolachlor to be extracted. After 1 day of mixing, 1 mL methanol samples were transferred into autosampler vials and stored until analysis to determine extracted pesticide concentration. Using this concentration, the concentration of pesticide associated with the ENM was calculated using eq. S7,[4]

$$C_{ENM} = \frac{C_{MeOH} * m_{MeOH}}{m_{ENM} * \rho_{MeOH}} \text{ (eq. S7)}$$

where C_{ENM} is the analyte concentration sorbed to the ENM at equilibrium, m_{ENM} is the total mass of ENM in the vial, m_{MeOH} is the mass of methanol used to extract the sorbed analyte from the ENM, C_{MeOH} is the measured analyte concentration in the methanol extract, and ρ_{MeOH} is the density of methanol. To determine the ENM-water equilibrium partitioning coefficient ($K_{\mathrm{ENM\text{-}W}}$ value), values of C_{ENM} and C_{W} were plotted, and a linear regression was performed using the software GraphPad Prism. The slope of the best-fit linear regression line was used to determine the $K_{\mathrm{ENM\text{-}W}}$ value according to eq. S7 (which is the ENM specific version of eq. S6).

Desorption Experiments

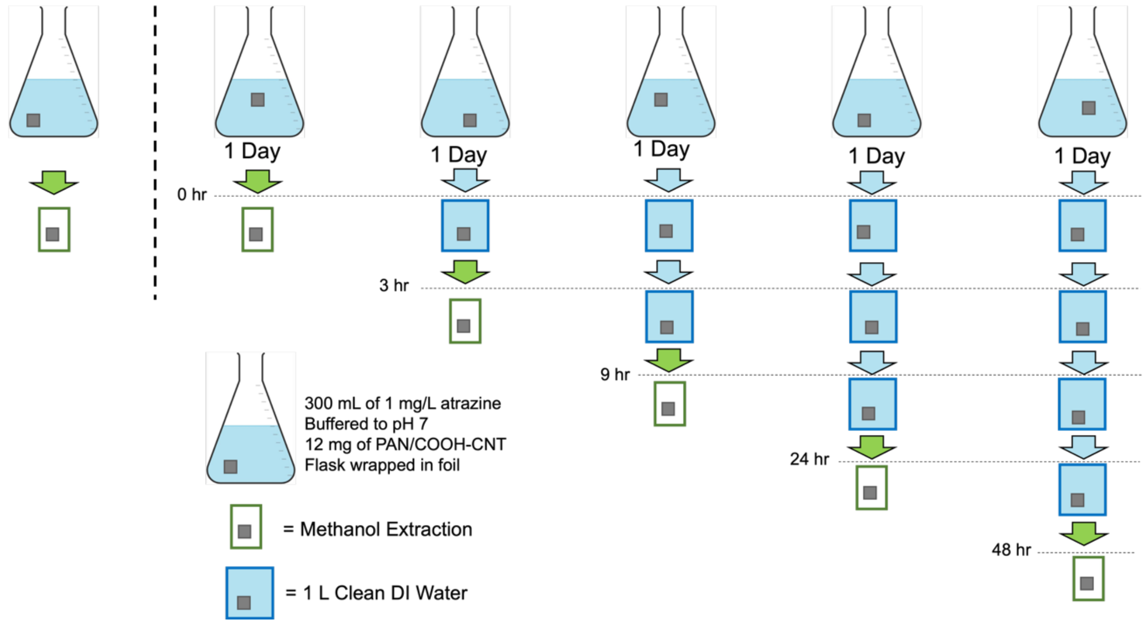

Figure S1. Experiment set-up and basic actions taken during the course of a sorption reversibility experiment. First, a set of identical ENMs was equilibrated with either a "high" or "low" concentration micropollutant solutions (1 mg/L and 5 µg/L, respectively) over 1 day. Next, equilibrated ENMs were transferred into separate 1 L bottles of DI water, with replicate reactors sacrificially sampled over the course of 48 hours. For analysis, samples were collected both of the aqueous phase and the ENM solid phase after methanol extraction, with samples and ENMs processed according to methods for sorption uptake experiments. Importantly, at each sampling point (typically a 3-24 h interval), the fresh DI water was exchanged in each of the remaining vials, simulating release of the bound micropollutant into an infinite sink.

Dynamic Micropollutant Uptake and Release Experiments

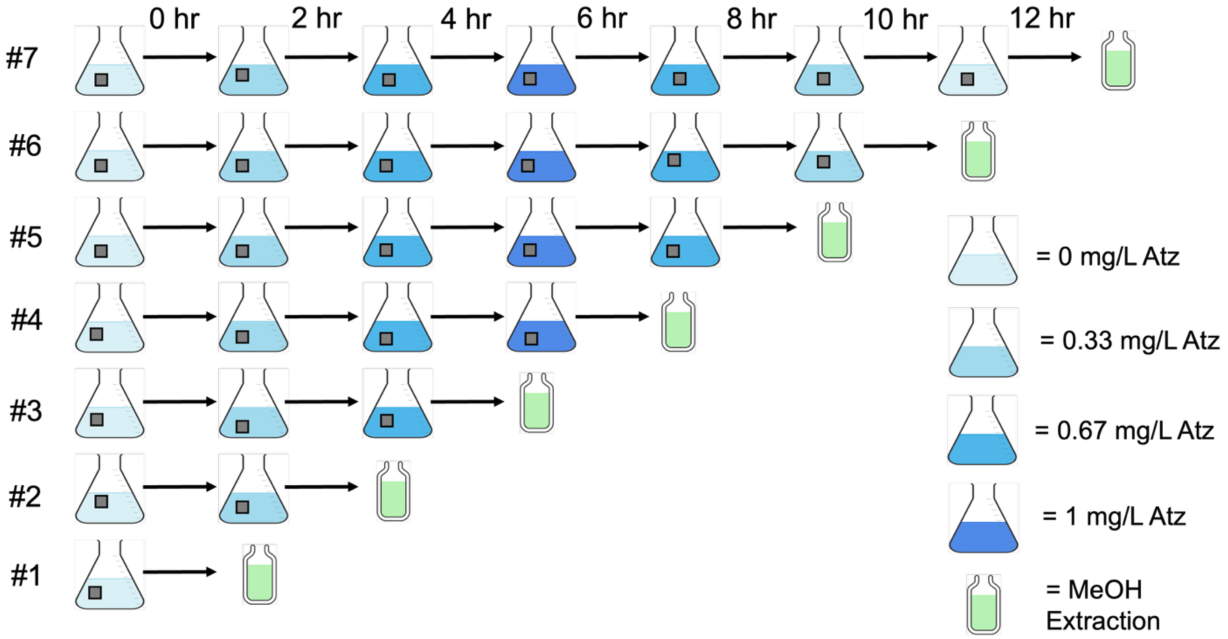

Figure S2. Experiment set-up and basic actions taken during the course of a sorption dynamic uptake and release experiment. Briefly, a set of identical ENMs (10 mg) were initially placed in 300 mL of DI water free of atrazine. Then, every 2 hours thereafter, a change in the aqueous phase concentration of atrazine was initiated by transferring each ENM in the set to a new atrazine-containing solution. Initially every two hours, the atrazine concentration was increased from 0.33 mg/L to 0.67 mg/L and then 1 mg/L, before subsequently decreasing the aqueous phase concentration every two hours to 0.67 mg/L, then 0.33 mg/L, and then finally completing the experiment by placing the ENM in fresh DI water (as at the start of the trial). At each atrazine concentration change, an ENM from the identical set of reactors was sacrificially sampled and processed using previously described methods.

Field Deployments of ENM Samplers

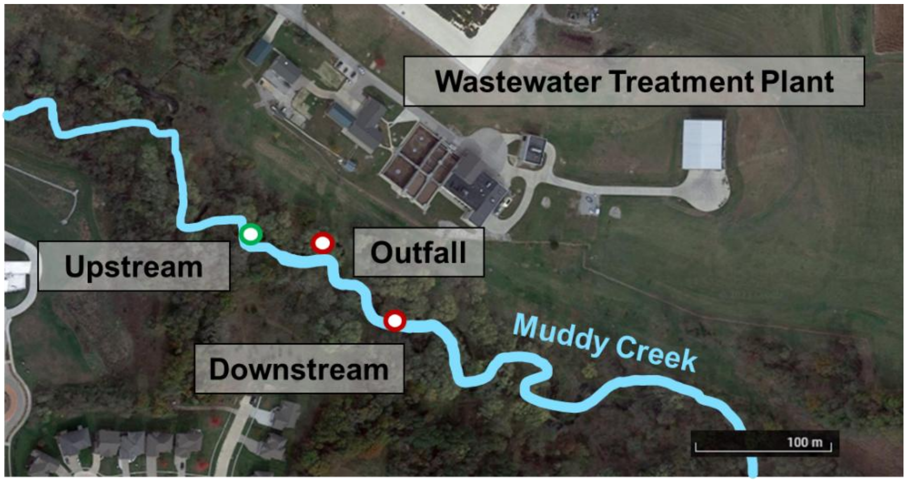

Figure S3. Satellite image of sampling location, Muddy Creek, in North Liberty, IA (41.723927, -91.592618). Samplers were deployed at the Upstream location, just upstream from the outfall of North Liberty wastewater treatment plant effluent. Image modified from satellite image obtained from Google Earth.[8]

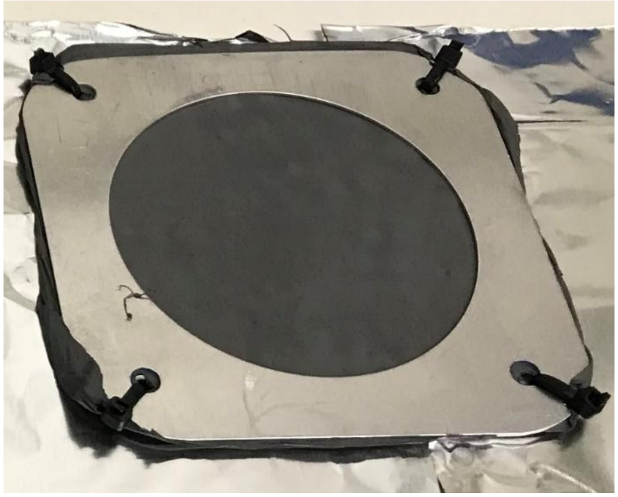

Figure S4. A close-up view of an ENM sampler. ENMs are sandwiched between two metal frames (9.5 cm x 9.5 cm), with an exposed area of 38.5 cm^2 and secured using zip ties. The dark gray color observed in the ENM suggests the incorporation of CNTs into the PAN matrix.

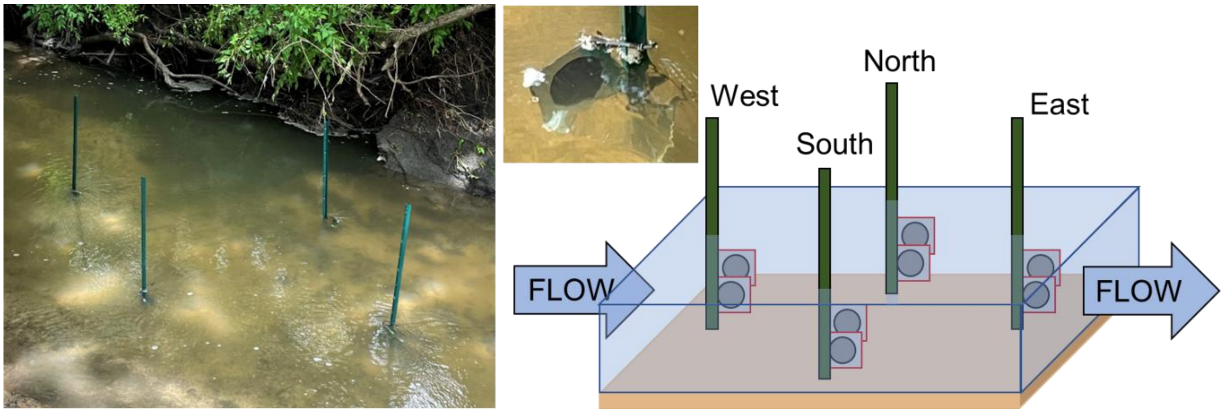

Figure S5. Field deployment configuration of ENM samplers in Muddy Creek (North Liberty, IA). ENM samplers were deployed in pairs at 4 locations within the creek as shown. ENM samplers were secured to 1.5 m metal garden stakes with zip ties, and the stakes were secured in the sediment of the creek so that the samplers were at middle depth in the creek. Flow at the sampling site flowed west to east (~0.29 m^3/s, USGS monitoring location 05454090), and 1 L grab samples were taken in amber glass bottles provided by Iowa State Hygienic Lab. Grab sample bottles were filled upstream (west) of the ENM samplers and emptied downstream (east) of the samplers three times before the actual sample was taken. ENMs serving as field blanks were taken on location but remained wrapped in aluminum foil until analysis in lab.

Analytical Methods

Table S5. Summary of LC-DAD Parameters for target analytes. Because each experiment was performed with each analyte individual, separate methods were used to analyze each analyte. Methods were adapted from previous work.[20]

Compound	Flow (mL/min)	Inj. Volume (µL)	Solvent A	Solvent B	%A	%B	Run Time (min)	RT (min)	Wavelength (nm)
Metolachlor	1	100	DI	ACN	40	60	9	7.2	220
Atrazine	1	100	DI	ACN	50	50	6	4.9	223
2,4-D	1.5	40	17.5 mM Acetic Acid	ACN	50	50	5	2.8	236
Diuron	1.2	40	DI	ACN	70	30	22	17.8	254

Table S6. Summary of LC-MS/MS Parameters. Methods adapted from USGS.[9, 10]

Mobile Phase Gradient			
Time	%A	%B	Flow
0	90	10	0.4
4	80	20	0.4
5	60	40	0.6
10	40	60	0.6
15	20	80	0.6
19	0	100	0.6
21	0	100	0.6
23	90	10	0.6

Mobile Phase Parameters	
Flow (mL/min)	0.4-0.6
Injection Volume (µL)	10
Solvent A	Water
Solvent B	Acetonitrile
Run Time (min)	22

Table S7. MS/MS Parameters. Methods adapted from USGS.[9, 10]

MS/MS Parameters	
MRM Mode	+
Gas Temperature (N_2, °C)	200
Gas Flow (L/min)	7
Nebulizer Pressure (psi)	20
Sheath Gas Temperature (°C)	250
Sheath Gas Flow (L/min	10
Capillary Volatage (+)/(-)	2500 / 2500
Nozzle Voltage (+)/(-)	2000 / 2000

Table S8. MRM Parameters. Methods adapted from USGS.[9, 10]

	MRM Transitions									
Compound	RT (min)	Parent Ion (m/z)	Quant Ion (m/z)	Quant CE (V)	Qual Ion (m/z)	Qual CE (V)	Fragment Voltage (V)	Dwell Time (ms)	Polarity	Cell Acceleration Voltage (V)
Metolachlor	18.2	284.1	252	10	176.1	25	60	200	+	3
Atrazine	14.2	216.1	174.1	20	146	20	120	200	+	3

Quality Assurance and Quality Control

Previous work demonstrated that recovery of analyte from ENMs is generally high (~98%) and as such recoveries in experiments in this chapter are assumed to have 100% recovery.[3, 4]

Table S9. Average field blank ENM concentration with standard deviation. Also listed is limit of quantification, LOQ, for ENM sampler concentration of each compound, at or below which (had it occurred) would not be reportable.

Compound	Average Conc. (ng/g)	LOQ (ng/g)
Atrazine	6.3 ± 1.1	7.7
Metolachlor	16.4 ± 12.2	33.3

ATR-FTIR Method

Fourier-transform infrared spectra were collected using a Thermo Scientific Nicolet iS50 FT-IR equipped with Pike Technologies single reflection diamond IRIS ATR. A background spectrum was collected prior to sample measurement and subtracted automatically in Thermo Nicolet OMNIC software (Thermo Scientific, Madison, WI, USA). Samples were clamped against the diamond crystal using the ATR sample clamp to maintain consistent pressure per sample. Spectra were recorded in the range of 4000 cm^{-1} to 650 cm^{-1} with a spectral resolution of 4 cm^{-1}, and 288 scans per sample.

Results & Discussion

Material Characterization

Table S10. Surface area, pore volume, and average nanofiber diameter of ENM synthesized herein. The (L) indicates samples that were processed via washing to removal any leachable SDS to exploit its role as a porogen.

Sorbent	Surface Area (m^2/g; *n*=6)	Pore Volume (μL/g; *n*=3)	Nanofiber Diameter (nm)
PAN	12 ± 0.5	23 ± 1.7	160 ± 30
PAN/NF-CNT_{20}	15 ± 2.4	37 ± 8.0	210 ± 50
PAN/$TBAB_{20}$/NF-CNT_{20}	14 ± 1.2	39 ± 9.0	240 ± 110
PAN/SDS_{20}/NF-CNT_{20}	14 ± 0.8	49 ± 2.7	245 ± 100
PAN/SDS_{20}/NF-CNT_{20} (L)	21 ± 2.2	66 ± 7.7	290 ± 110
PAN/COOH-CNT_{20}	17 ± 2.0	43 ± 6.0	200 ± 40
PAN/$TBAB_{20}$/COOH-CNT_{20}	18 ± 2.4	44 ± 6.3	240 ± 130
PAN/SDS_{20}/COOH-CNT_{20}	14 ± 1.9	49 ± 4.8	280 ± 100
PAN/SDS_{20}/COOH-CNT_{20} (L)	33 ± 3.0	103 ± 12	310 ± 140

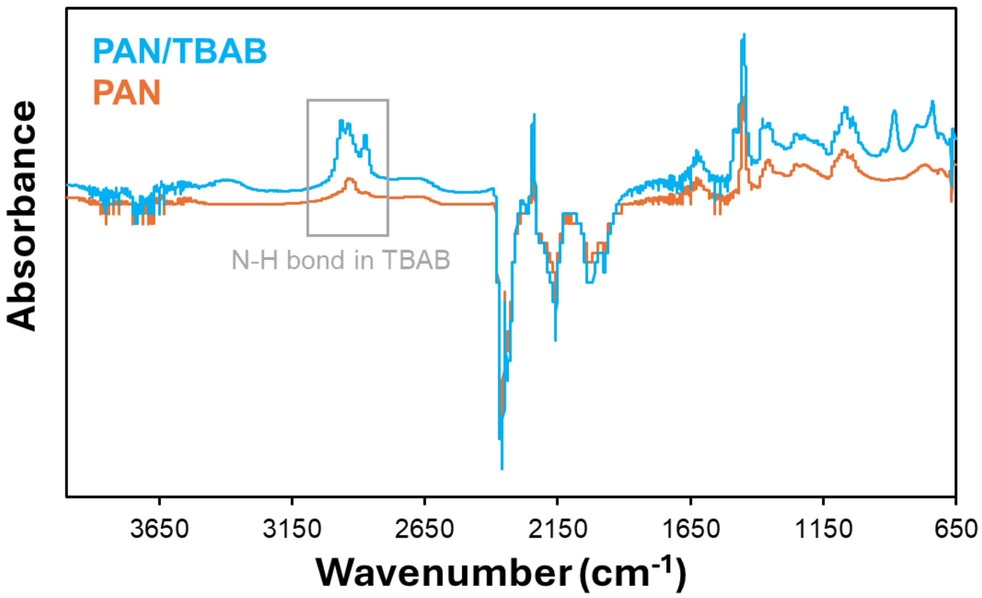

Figure S6. ATR-FTIR spectra for PAN and PAN/TBAB, with the region indicative of the N-H bond in TBAB noted.

(a)

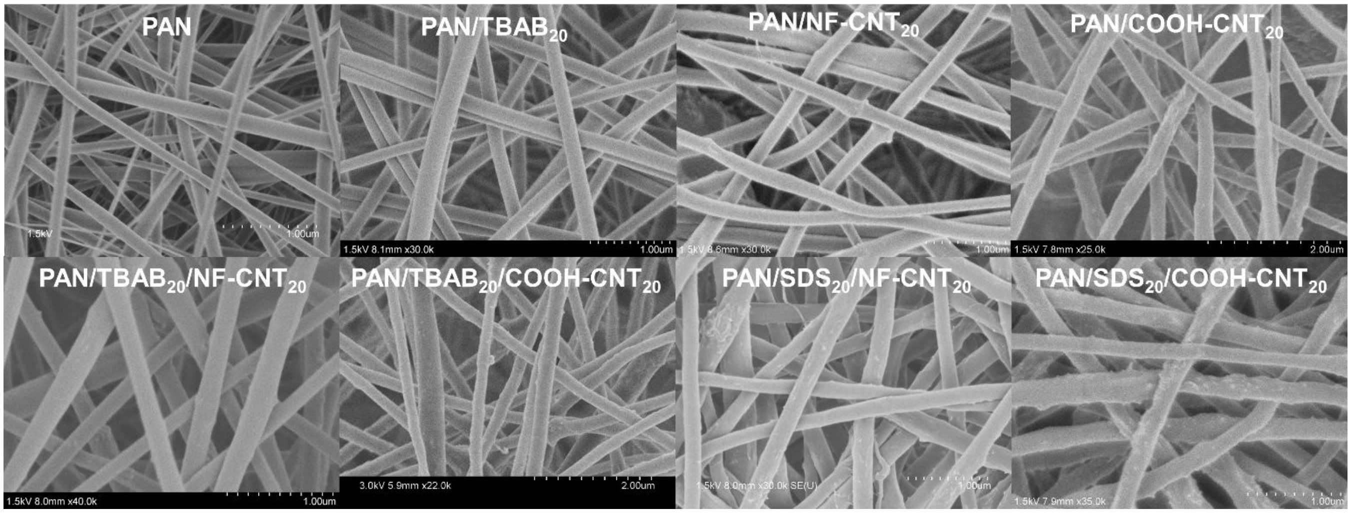

(b)

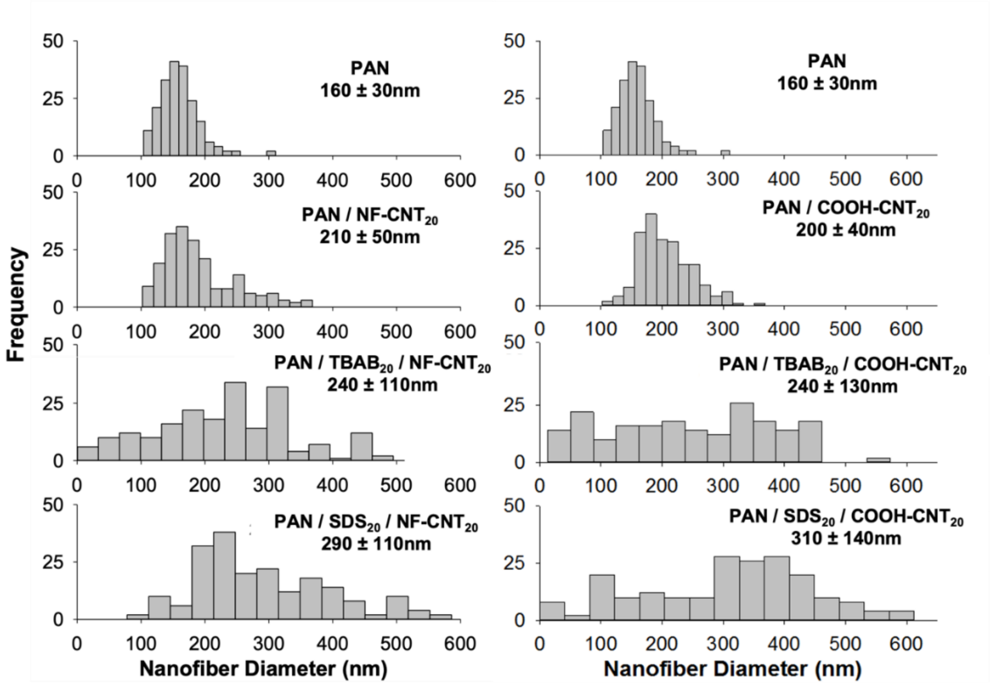

Figure S7. (a) Representative SEM images of various ENM formulations as indicated. (b) Histograms of nanofibers within various ENM formulations. Histograms represent diameter measurements of at least 200 or more nanofibers in the program ImageJ. ENM formulation, average nanofiber diameter and standard deviation are provided.

ENM Uptake, Release and Dynamic Modeling Results

We observed good agreement between the measured and modeled data for all three model systems, with an average R^2 of 0.93 (± 0.10). The only exception was PAN with atrazine, which yielded an R^2 of 0.39 due to an unexpectedly high sorption value (approximately three times higher than the others) at the first time point (**Fig. 1b**). The MSE values were also low, with a maximum value of 0.07. Further comparison between the experimentally measured and model-predicted $K_{ENM–W}$ values yielded an R^2 of 0.99 for the uptake experiments and 0.97 for the comparison of f values from the release experiments. In the dynamic experiments, $K_{ENM–W}$ and f could be compared, whereas the kinetic parameters could not be directly compared due to differences in mixing conditions relative to the uptake and release experiments. Here, the $K_{ENM–W}$ comparison yielded an R^2 of 0.51, mainly driven by the difference between the PAN/$TBAB_{20}$/COOH–CNT_{20} values ($K_{ENM–W,obs}$ = 0.07 and $K_{ENM–W,mod}$ = 0.11), while f showed an R^2 of 0.98. Overall, these results demonstrate that the selected model describes the behavior of the ENMs and associated chemicals well, while providing meaningful equilibrium and kinetic parameters. All resulting parameters are presented in detail in **Tables S11–S13**.

Table S11. Summary of equilibrium partition coefficients and kinetic parameters from uptake experiments. Values were derived from modeling data presented in **Figs. 1-2** of the main text. Modeled values represent the mean ± standard error, computed using the 'jackknife' method. [3, 7]

Material	Chemical	Mixing condition	K_{ENM-W} (L/g) [a]	K_{ENM-W} (L/g)	k_e (1/d)	$t_{90\%}$ (d)	R^2	MSE	Fig
PAN	2,4-D	w/o mixing	0.015 ± 0.002	0.014 ± 0.002	4.61 ± 1.93	0.52 ± 0.22	0.814	0.000	1a
PAN/$TBAB_{20}$	2,4-D	w/o mixing	0.659 ± 0.02	0.654 ± 0.011	5.39 ± 0.39	0.43 ± 0.03	0.996	0.011	1a
PAN/NF-CNT_{20}	2,4-D	w/o mixing	0.029 ± 0.002	0.027 ± 0.006	8.39 ± 13.08	0.33 ± 0.38	0.731	0.001	1a
PAN/COOH-CNT_{20}	2,4-D	w/o mixing	0.064 ± 0.005	0.066 ± 0.003	11.03 ± 1.93	0.21 ± 0.03	0.950	0.001	1a
PAN/$TBAB_{20}$/NF-CNT_{20}	2,4-D	w/o mixing	0.657 ± 0.04	0.648 ± 0.037	4.38 ± 2.02	0.54 ± 0.22	0.971	0.068	1a
PAN/$TBAB_{20}$/COOH-CNT_{20}	2,4-D	w/o mixing	0.589 ± 0.07	0.569 ± 0.047	5.59 ± 1.35	0.41 ± 0.10	0.959	0.067	1a
PAN	atrazine	w/o mixing	0.0024 ± 0.0003	0.003 ± 0.001	23.48 ± 43.97	0.14 ± 0.11	0.384	0.000	1b
PAN/NF-CNT_{20}	atrazine	w/o mixing	0.032 ± 0.003	0.030 ± 0.002	8.16 ± 2.88	0.29 ± 0.10	0.938	0.000	1b
PAN/COOH-CNT_{20}	atrazine	w/o mixing	0.067 ± 0.002	0.066 ± 0.002	6.50 ± 0.67	0.35 ± 0.04	0.992	0.000	1b
PAN/$TBAB_{20}$/NF-CNT_{20}	atrazine	w/o mixing	0.028 ± 0.001	0.026 ± 0.001	13.05 ± 5.36	0.18 ± 0.08	0.886	0.000	1b
PAN/$TBAB_{20}$/COOH-CNT_{20}	atrazine	w/o mixing	0.066 ± 0.001	0.064 ± 0.002	15.57 ± 3.49	0.15 ± 0.04	0.968	0.000	1b
PAN/SDS_{20}/NF-CNT_{20}	atrazine	w/o mixing	0.051 ± 0.003	0.048 ± 0.003	60.14 ± 29.31	0.04 ± 0.03	0.873	0.001	2a
PAN/SDS_{20}/COOH-CNT_{20}	atrazine	w/o mixing	0.097 ± 0.002	0.094 ± 0.002	12.37 ± 2.61	0.19 ± 0.04	0.978	0.001	2a
PAN	metolachlor	w/o mixing	0.0106 ± 0.0004	0.010 ± 0.000	b	b	0.993	0.000	1c
PAN/NF-CNT_{20}	metolachlor	w/o mixing	0.029 ± 0.003	0.029 ± 0.002	7.99 ± 2.46	0.29 ± 0.09	0.941	0.000	1c
PAN/COOH-CNT_{20}	metolachlor	w/o mixing	0.053 ± 0.005	0.051 ± 0.003	8.54 ± 2.33	0.27 ± 0.07	0.957	0.001	1c
PAN/$TBAB_{20}$/NF-CNT_{20}	metolachlor	w/o mixing	0.072 ± 0.003	0.063 ± 0.005	79.85 ± 45.96	0.03 ± 0.03	0.844	0.003	1c
PAN/$TBAB_{20}$/COOH-CNT_{20}	metolachlor	w/o mixing	0.088 ± 0.002	0.082 ± 0.003	24.54 ± 7.13	0.09 ± 0.03	0.906	0.004	1c
PAN/SDS_{20}/NF-CNT_{20}	metolachlor	w/o mixing	0.088 ± 0.002	0.080 ± 0.006	17.01 ± 8.52	0.14 ± 0.07	0.874	0.006	2b
PAN/SDS_{20}/COOH-CNT_{20}	metolachlor	w/o mixing	0.089 ± 0.008	0.084 ± 0.005	30.00 ± 8.04	0.08 ± 0.02	0.915	0.004	2b
PAN	diuron	w/o mixing	0.015 ± 0.002	0.016 ± 0.001	13.88 ± 43.86	0.39 ± 0.40	0.925	0.000	1d

PAN/SDS_{20}	diuron	w/o mixing	0.061 ± 0.005	0.055 ± 0.004	33.81 ± 27.28	0.07 ± 0.05	0.785	0.003	-
PAN/NF-CNT_{20}	diuron	w/o mixing	0.11 ± 0.01	0.106 ± 0.009	5.15 ± 1.47	0.45 ± 0.12	0.953	0.003	1d
PAN/COOH-CNT_{20}	diuron	w/o mixing	0.21 ± 0.02	0.213 ± 0.011	4.70 ± 0.60	0.49 ± 0.06	0.989	0.003	1d
PAN/$TBAB_{20}$/NF-CNT_{20}	diuron	w/o mixing	0.29 ± 0.01	0.276 ± 0.017	14.02 ± 3.86	0.17 ± 0.05	0.946	0.019	1d
PAN/$TBAB_{20}$/COOH-CNT_{20}	diuron	w/o mixing	0.43 ± 0.02	0.409 ± 0.019	10.33 ± 3.58	0.23 ± 0.08	0.953	0.041	1d
PAN/SDS_{20}/NF-CNT_{20}	diuron	w/o mixing	0.17 ± 0.01	0.161 ± 0.012	9.86 ± 1.71	0.23 ± 0.04	0.943	0.007	2c
PAN/SDS_{20}/COOH-CNT_{20}	diuron	w/o mixing	0.35 ± 0.01	0.333 ± 0.016	6.25 ± 2.17	0.37 ± 0.12	0.968	0.024	2c

[a] Corresponds to the mean ± standard deviation of the last three laboratory measurements in each experiment (e.g., days 1, 1.5, and 2). [b] Uptake of metolachlor into PAN was too rapid to model k_e due to insufficient early-time data.

Table S12. Summary of kinetic parameters and fraction of atrazine retained by PAN from release experiments, derived from modeling data presented in **Fig. 3** of the main text. Modeled values represent the mean ± standard error, computed using the 'jackknife' method. [3, 7]

Material	Chemical	Mixing condition	f [a] (%)	f (%)	k_e (1/d)	$t_{90\%}$ (d)	R^2	MSE	Fig
PAN/COOH-CNT_{20}	atrazine	w/ mixing (50 rpm)	32 ± 4	30.2 ± 3.6	2.85 ± 1.01	0.83 ± 0.28	0.98	0.00	3ad
PAN/$TBAB_{20}$/COOH-CNT_{20}	atrazine	w/ mixing (50 rpm)	18 ± 2	20.0 ± 4.7	9.96 ± 4.92	0.25 ± 0.19	0.98	0.00	3ad
PAN/SDS_{20}/COOH-CNT_{20}	atrazine	w/ mixing (50 rpm)	14 ± 4	17.7 ± 6.2	11.72 ± 7.25	0.23 ± 0.25	0.98	0.00	3ad
PAN/COOH-CNT_{20} [b]	atrazine	w/ mixing (125 rpm)	29 ± 9	28.5 ± 12.3	3.80 ± 2.85	0.71 ± 0.56	0.93	0.01	3b
PAN/COOH-CNT_{20} [b]	atrazine	w/ mixing (50 rpm)	32 ± 4	30.2 ± 3.6	2.85 ± 1.01	0.83 ± 0.28	0.98	0.00	3b
PAN/COOH-CNT_{20} [b]	atrazine	w/o mixing	37 ± 2	38.3 ± 4.4	5.65 ± 2.36	0.43 ± 0.23	0.97	0.00	3b
PAN/COOH-CNT_{20} [c]	atrazine	w/ mixing (125 rpm)	31 ± 3	31.5 ± 3.5	4.34 ± 1.05	0.54 ± 0.13	0.99	0.00	3b
PAN/COOH-CNT_{20} [c]	atrazine	w/ mixing (50 rpm)	44 ± 5	45.6 ± 8.7	5.10 ± 3.90	0.54 ± 0.49	0.92	0.00	3b
PAN/COOH-CNT_{10}	atrazine	w/ mixing (50 rpm)	23 ± 5	23.9 ± 6.7	5.02 ± 2.27	0.49 ± 0.26	0.97	0.00	3d
PAN/SDS_{20}/COOH-CNT_{10}	atrazine	w/ mixing (50 rpm)	6 ± 2	9.2 ± 6.7	14.04 ± 9.58	0.21 ± 0.26	0.98	0.00	3d & S8a
PAN/SDS_{20}/COOH-CNT_{10}	metolachlor	w/ mixing (50 rpm)	8 ± 0.4	9.4 ± 1.8	16.14 ± 7.97	0.16 ± 0.12	0.99	0.00	S8a

[a] Corresponds to the mean ± standard deviation of the last two laboratory measurements in each experiment. [b] With exchange of clean water ("Refreshing water"). [c] Without exchange of clean water.

Table S13. Summary of kinetic parameters and fraction of atrazine retained by PAN from dynamic step experiments, derived from modeling data presented in **Fig. 4** of the main text. Modeled values represent the mean ± standard error, computed using the 'jackknife' method. [3, 7]

**Material**	**Chemical**	**Mixing**	**f [a] (%)**	**f (%)**	k_e **(1/d)**	k_u **(L/g/d)**	K_{ENM-W} **(L/g)** [b]	K_{ENM-W} **(L/g)**	R^2	**MSE**	**Fig**
PAN/COOH-CNT_{20}	atrazine	w/ mixing (125 rpm)	30 ± 6 [c]	32.9 ± 2.4	7.48 ± 1.38	0.47 ± 0.06	0.0667	0.063 ± 0.005	0.9799	0.0000	4
PAN/$TBAB_{20}$/COOH-CNT_{20}	atrazine	w/ mixing (125 rpm)	18 ± 2	20.1 ± 0.6	9.91 ± 1.57	1.11 ± 0.18	0.066	0.112 ± 0.007	0.9655	0.0001	4
PAN/SDS_{20}/COOH-CNT_{20}	atrazine	w/ mixing (125 rpm)	14 ± 4	20.3 ± 0.8	10.98 ± 2.23	1.21 ± 0.23	0.097	0.110 ± 0.009	0.9421	0.0001	4
PAN/SDS_{20}/COOH-CNT_{10}	atrazine	w/ mixing (125 rpm)	6 ± 2	10.2 ± 0.1	9.77 ± 0.80	0.39 ± 0.04	0.052[d]	0.040 ± 0.002	0.9745	0.0000	4

[a] Corresponds to the values obtained via measurements from the release experiments, **Table S12**. [b] Corresponds to the measured values obtained from the uptake experiments (**Table S11**). [c] Represents the average of PAN/COOH-CNT_{20}[b] and PAN/COOH-CNT_{20}[c] with mixing at 125 rpm. [d] Value obtained from the sorption isotherm, **Fig. S8b**.

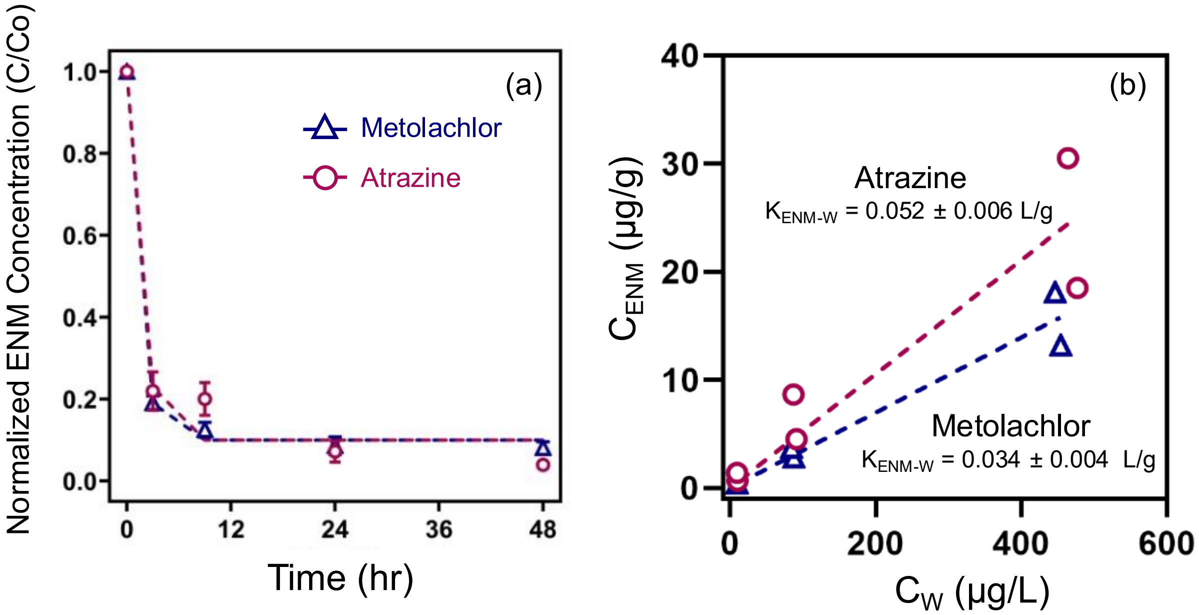

Figure S8. (a) Normalized analyte ENM concentration over time for PAN /SDS_{20}/COOH-CNT_{10} previously equilibrated with 1 mg/L analyte solution and then placed in 1 L of clean, mixed, DI water. Release data are shown as a function of the concentration of the metolachlor measured in the ENM at each time point normalized to the concentration of metolachlor measured in the ENM at the start of the release experiment (i.e., the amount of metolachlor bound to the ENM after equilibration with a 1 mg/L metolachlor solution). For comparison, we present data for the desorption results for atrazine from the same ENM formulation under the same experimental conditions (also shown in Fig. 3d). For these experiments, the DI water was refreshed at every sacrificial sampling time point (3, 9, and 24 h) to approximate an infinite sink. Modeled parameters, R^2 and MSE are presented in **Table S12**. Dashed lines correspond to the model described by Eq. (2). (b) Low concentration sorption isotherm for PAN /SDS_{20}/COOH-CNT_{10} with atrazine and metolachlor. Experimental conditions: 10 mg of ENM (final loading of 1.25 g/L), mixed end-over-end, initial analyte concentrations of 10, 100, and 500 µg/L, pH 7 buffered by 5 mM potassium phosphate monobasic buffer). Dashed lines represent linear regressions analyses from which $K_{ENM\text{-}W}$ values were determined.

Table S14. Results from ENM and grab sample analysis. Grab sample analysis was performed at the Iowa State Hygienic lab using EPA Method 8270, LOQ = 100 ng/L. Reported aqueous atrazine concentrations are the aqueous concentrations calculated from the measured ENM atrazine concentration via methanol extraction assuming a 100% extraction recovery of analyte from the ENM sampler. All ENM samplers were above the LOQ, 7.7 ng/g.

	Grab Sample (ng/L)	Reported Aqueous Atrazine Concentration (ng/L)				Measured ENM Atrazine Concentration (ng/g)			
Deployment Start	600	West	East	South	North	West	East	South	North
End of Round 1	600	434	522	634	424	21.7	26.1	31.7	21.2
End of Round 2	600	496	366	418	356	24.8	18.3	20.9	17.8

Table S15. Results from ENM and grab sample analysis. Grab sample analysis was performed at the Iowa State Hygienic lab using EPA Method 8270, LOQ = 100 ng/L. Reported aqueous metolachlor concentrations are the aqueous concentrations calculated from the measured ENM metolachlor concentration via methanol extraction assuming a 100% extraction recovery of analyte from the ENM sampler. All ENM samplers were above the LOQ, 33.3 ng/g.

	Grab Sample (ng/L)	Reported Aqueous Metolachlor Concentration (ng/L)				Measured ENM Metolachlor Concentration (ng/g)			
Deployment Start	1500	West	East	South	North	West	East	South	North
End of Round 1	1700	7206	8300	9715	9253	245	282	330	315
End of Round 2	1900	8765	6921	7029	6418	298	235	239	218

References

(1) Peter, K. T.; Johns, A. J.; Myung, N. V.; Cwiertny, D. M. Functionalized polymer-iron oxide hybrid nanofibers: Electrospun filtration devices for metal oxyanion removal. *Water Research* **2017**, *117*, 207-217. DOI: https://doi.org/10.1016/j.watres.2017.04.007.
(2) Kim, S.; Chen, J.; Cheng, T.; Gindulyte, A.; He, J.; He, S.; Li, Q.; Shoemaker, B. A.; Thiessen, P. A.; Yu, B.; Zaslavsky, L.; Zhang, J.; Bolton, E. E. PubChem in 2021: new data content and improved web interfaces. *Nucleic Acids Research* **2020**, *49* (D1), D1388-D1395. DOI: 10.1093/nar/gkaa971 (acccessed 7/17/2025).
(3) Qian, J.; Jennings, B.; Cwiertny, David M.; Martinez, A. Emerging investigator series: development and application of polymeric electrospun nanofiber mats as equilibrium-passive sampler media for organic compounds. *Environmental Science: Processes & Impacts* **2017**, *19* (11), 1445-1456, 10.1039/C7EM00289K. DOI: 10.1039/C7EM00289K.
(4) Qian, J.; Martinez, A.; Marek, R. F.; Nagorzanski, M. R.; Zhi, H.; Furlong, E. T.; Kolpin, D. W.; LeFevre, G. H.; Cwiertny, D. M. Polymeric Nanofiber-Carbon Nanotube Composite Mats as Fast-Equilibrium Passive Samplers for Polar Organic Contaminants. *Environmental Science & Technology* **2020**, *54* (11), 6703-6712. DOI: 10.1021/acs.est.0c00609.
(5) Witkowski, P. J.; Jaffé, P. R.; Ferrara, R. A. Sorption and desorption dynamics of aroclor 1242 to natural sediment. *Journal of Contaminant Hydrology* **1988**, *2* (3), 249-269. DOI: https://doi.org/10.1016/0169-7722(88)90025-3.
(6) Ghosh, U.; Weber, A. S.; Jensen, J. N.; Smith, J. R. Congener level PCB desorption kinetics of field-contaminated sediments. *Journal of Soil Contamination* **1999**, *8* (5), 593-613.
(7) Caceci, M. S. Estimating error limits in parametric curve fitting. *Analytical Chemistry* **1989**, *61* (20), 2324-2327. DOI: 10.1021/ac00195a023.
(8) U.S. GS. *05454090 Muddy Creek at Coralville, IA.* *https://doi.org/10.5066/F7P55KJN. (Site information directly accessible at https://waterdata.usgs.gov/nwis/inventory/?site_no=05454050&agency_cd=USGS&amp*. (accessed 2025 June).
(9) Klarich, K. L.; Pflug, N. C.; DeWald, E. M.; Hladik, M. L.; Kolpin, D. W.; Cwiertny, D. M.; LeFevre, G. H. Occurrence of Neonicotinoid Insecticides in Finished Drinking Water and Fate during Drinking Water Treatment. *Environmental Science & Technology Letters* **2017**, *4* (5), 168-173. DOI: 10.1021/acs.estlett.7b00081.
(10) Klarich Wong, K. L.; Webb, D. T.; Nagorzanski, M. R.; Kolpin, D. W.; Hladik, M. L.; Cwiertny, D. M.; LeFevre, G. H. Chlorinated Byproducts of Neonicotinoids and Their Metabolites: An Unrecognized Human Exposure Potential? *Environmental Science & Technology Letters* **2019**, *6* (2), 98-105. DOI: 10.1021/acs.estlett.8b00706.
